# Supplementary material for: Resilience of the Eastern African electricity sector to climate driven changes in hydropower generation
Source: Nat Commun. 2019 Jan 17;10:302. doi: 10.1038/s41467-018-08275-7 (PMC6336820; doi:10.1038/s41467-018-08275-7)
Supplement: Supplementary file 1 — Supplementary Information [file 41467_2018_8275_MOESM1_ESM.pdf]

Supplementary material

Resilience of the Eastern African electricity sector to climate driven changes in hydropower generation

V.Sridharan et al.

Supplementary Figures 1-14: Installed capacity and electricity generation mix for analysed climate futures

The following figures illustrate the perfect foresight (PF) adaptation strategies for all the EAPP countries—across the different analysed climates. Both the capacity and generation mix are provided. Each page either has the capacity/generation mix for the reported GCM-emission scenario mix.

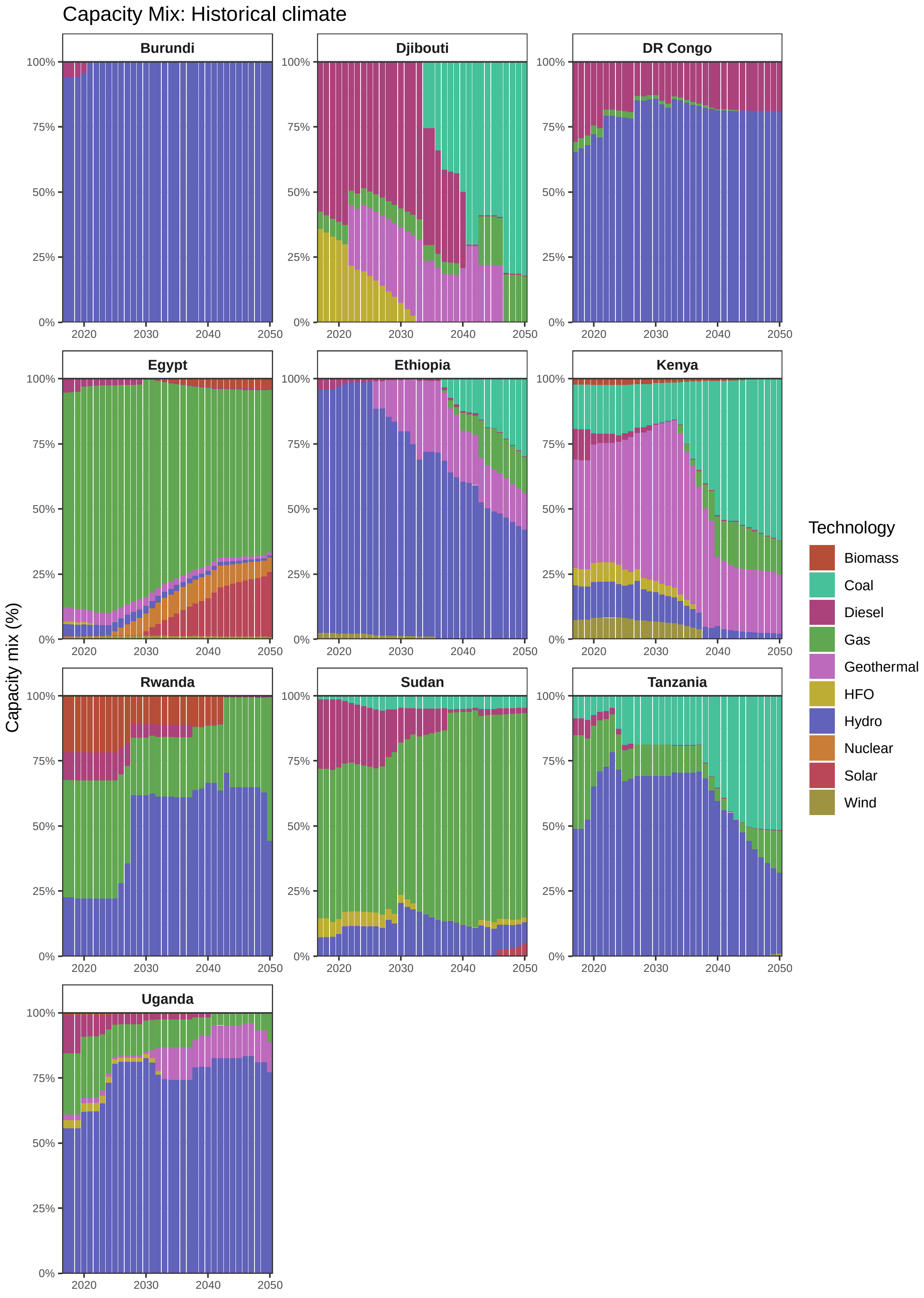

Supplementary Figure 1: Capacity mix for the baseline with historical climate trends

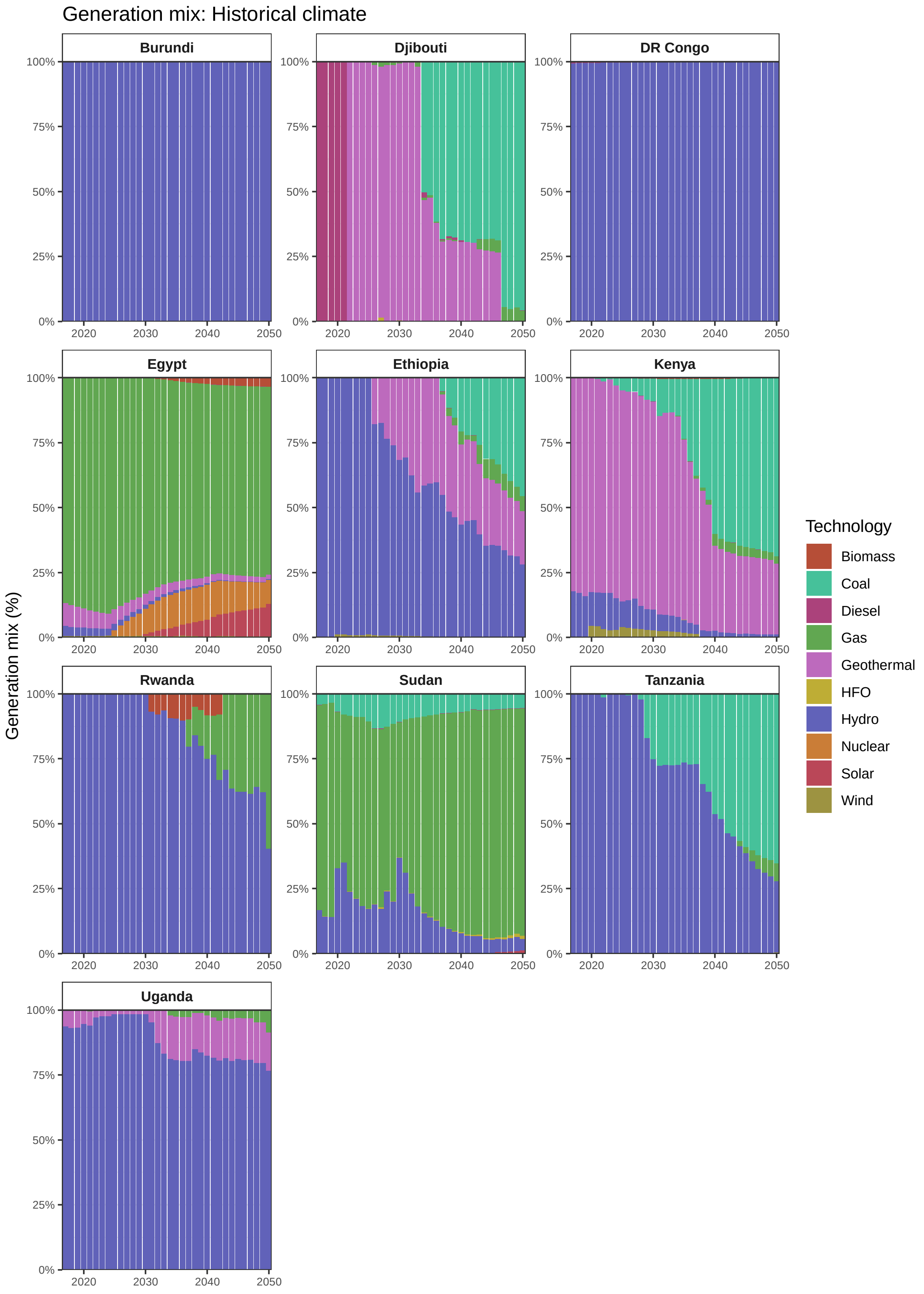

Supplementary Figure 2: Electricity generation mix for the baseline with historical climate trends

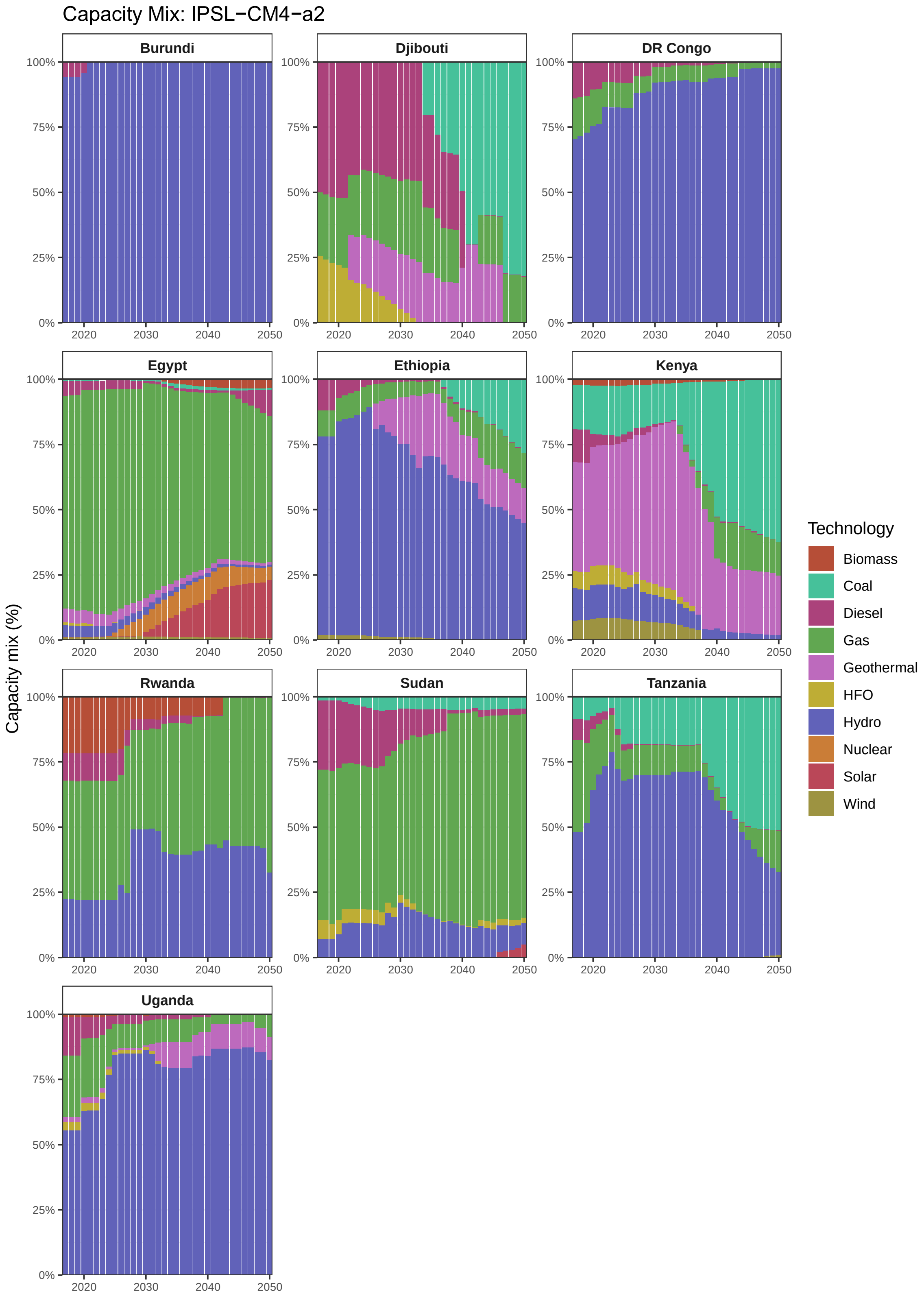

Supplementary Figure 3: Capacity mix. GCM: IPSL-CM4, Scenario: a2

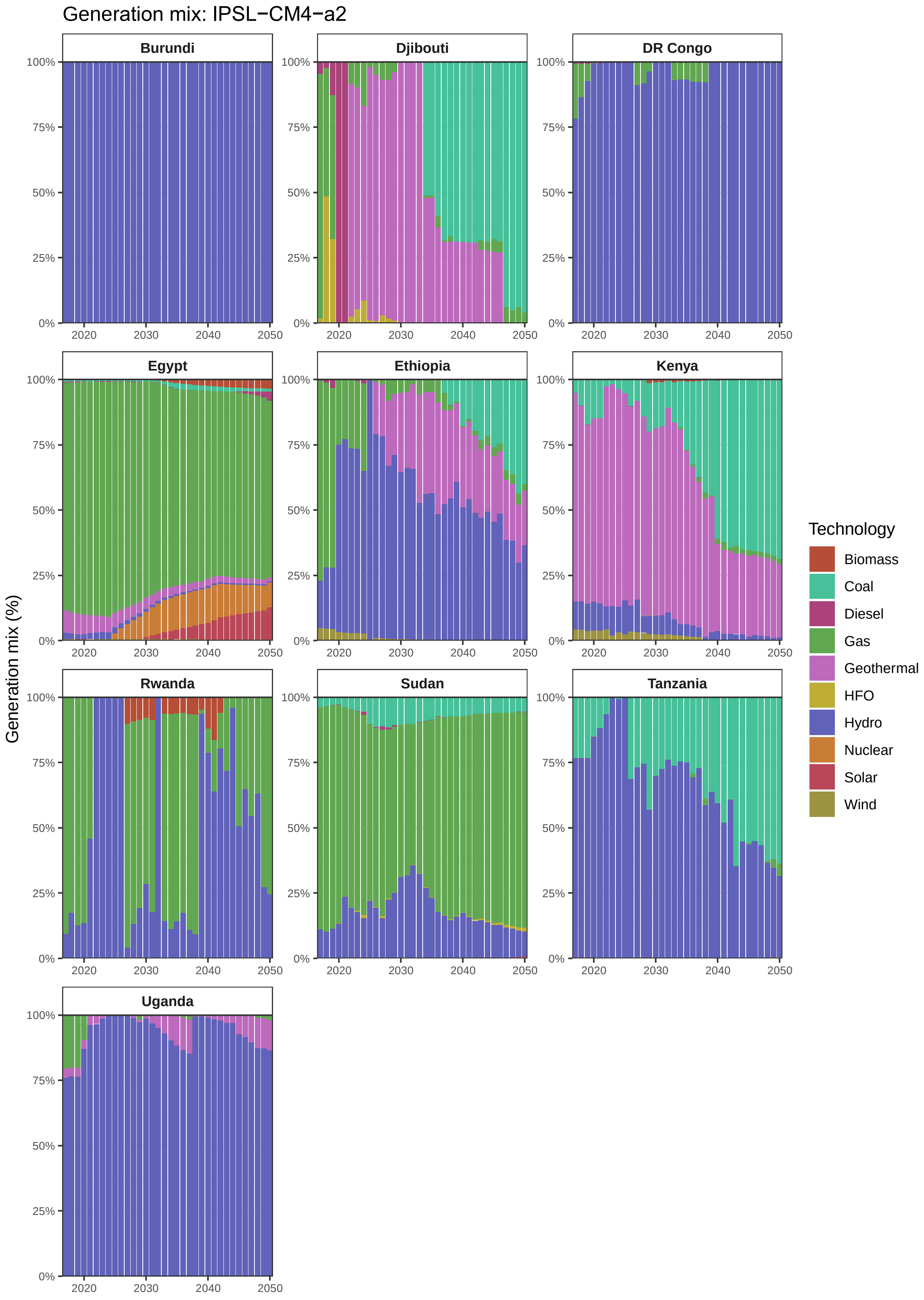

Supplementary Figure 4: Electricity generation mix. GCM: IPSL-CM4, Scenario: a2

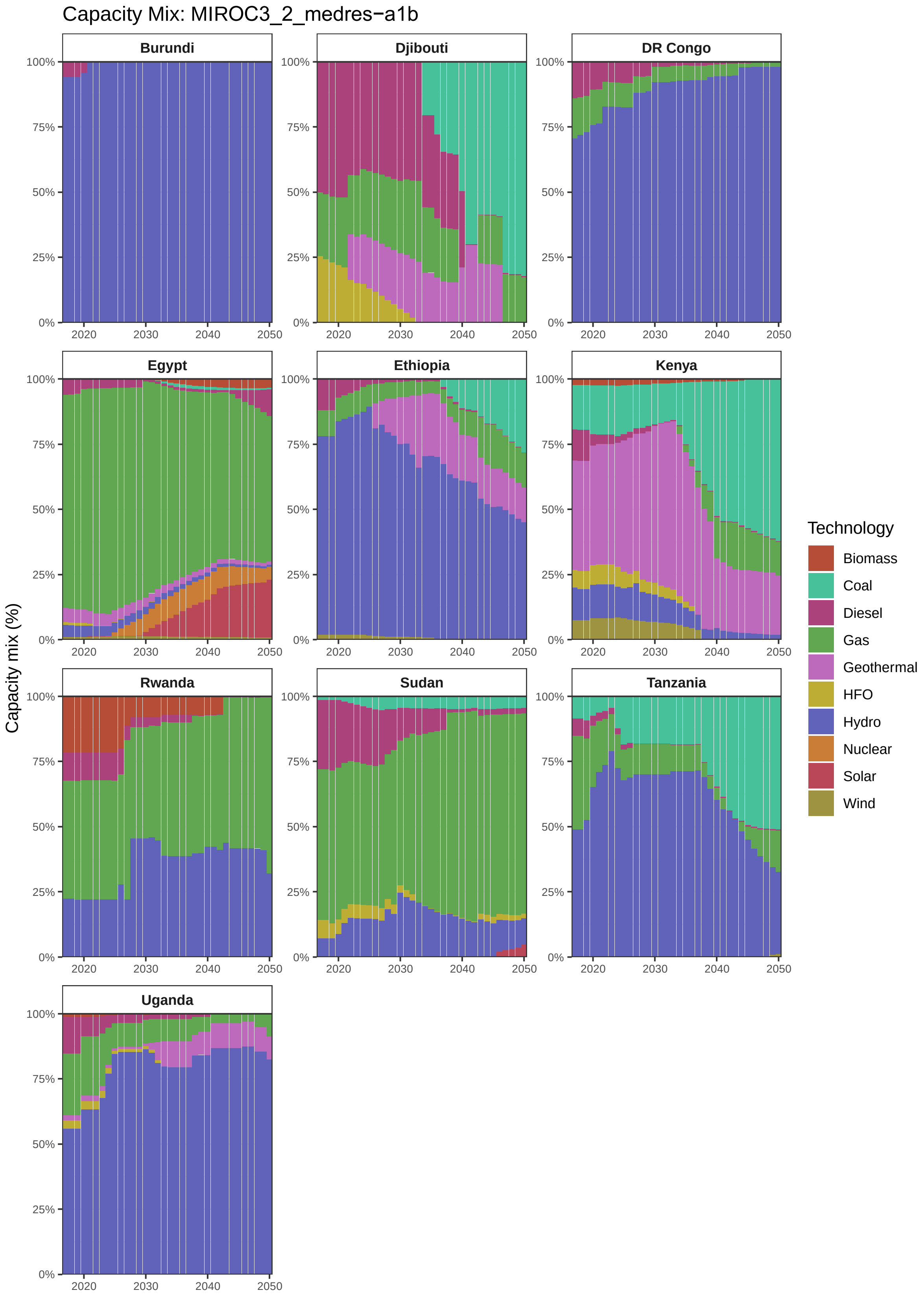

Supplementary Figure 5: Capacity mix. GCM: MIROC3_2_medres, Scenario: a1b

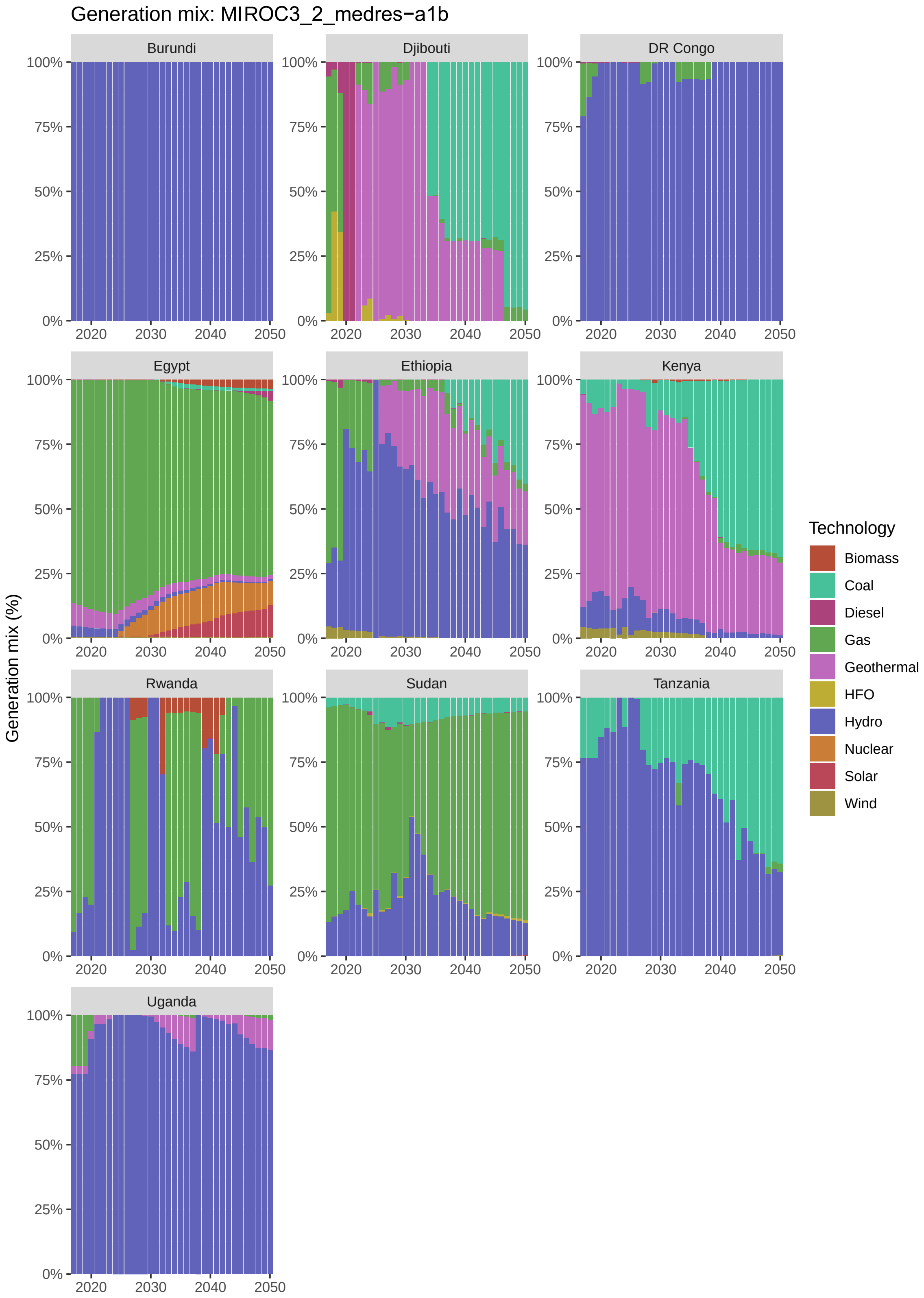

Supplementary Figure 6: Electricity generation mix, GCM: MIROC3_2_medres, Scenario: a1b

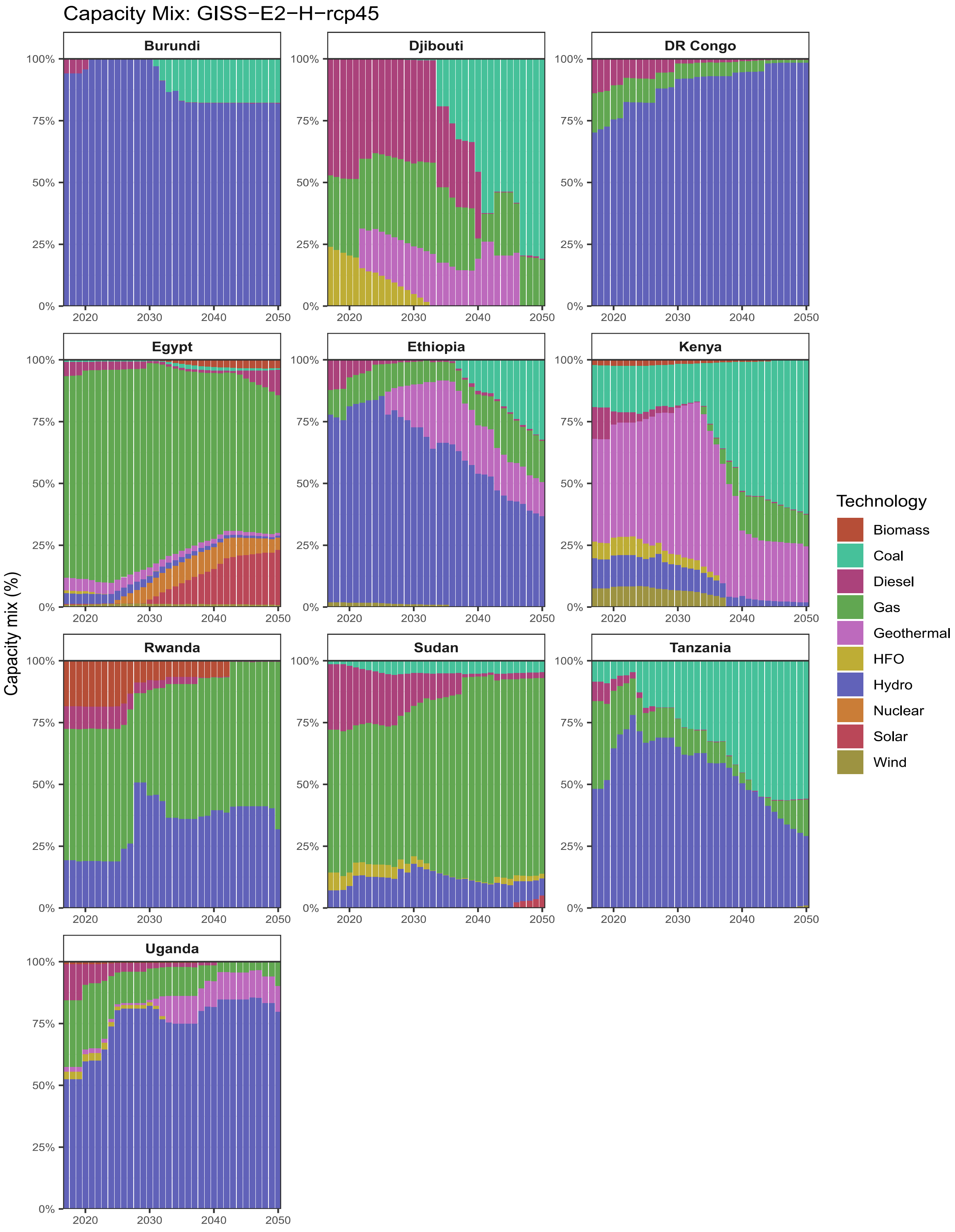

Supplementary Figure 7: Capacity mix. GCM: GISS-E2-H, Scenario: RCP45

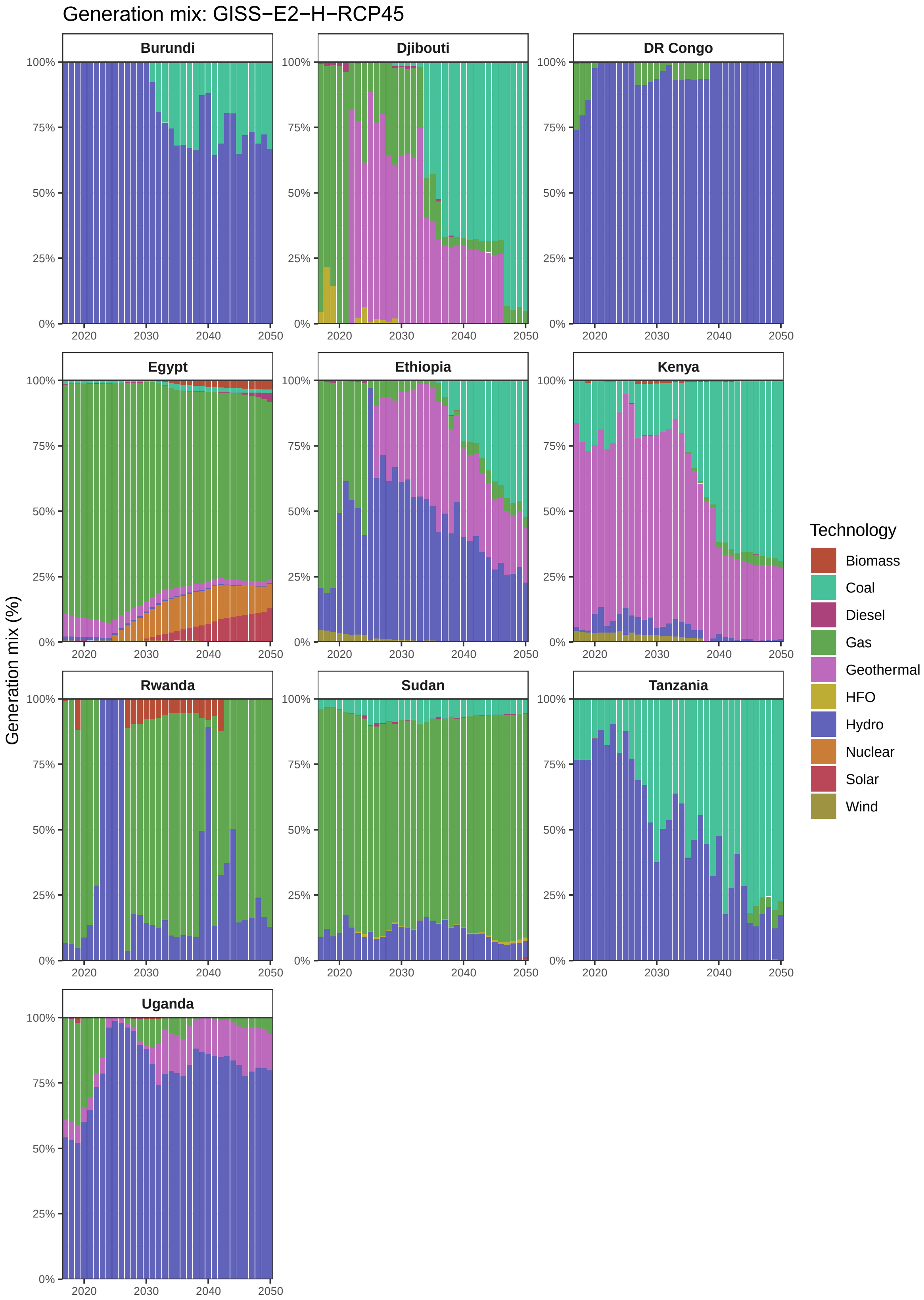

Supplementary Figure 8: Electricity generation mix, GCM: GISS-E2-H, Scenario: RCP45

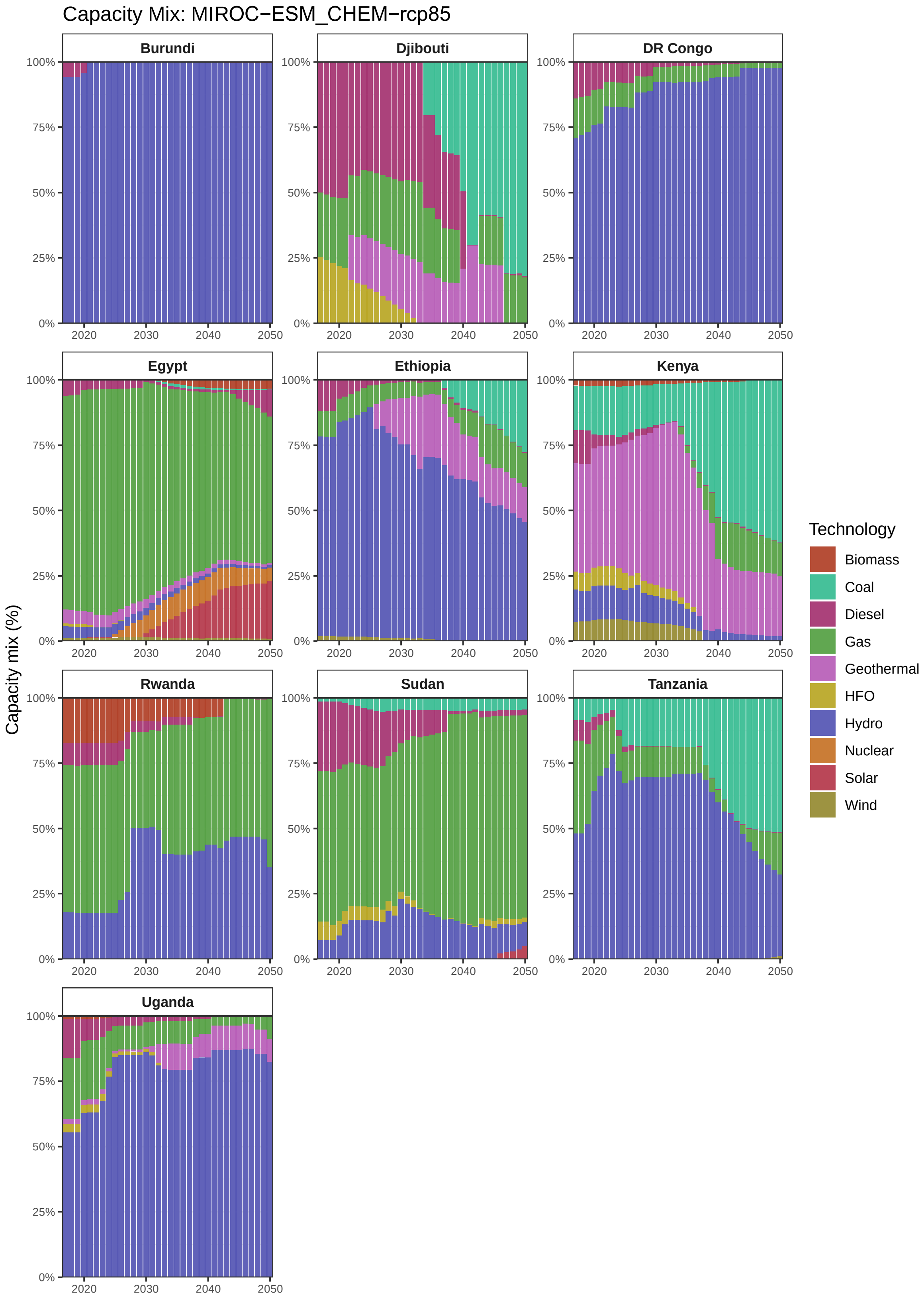

Supplementary Figure 9: Capacity mix. GCM: MIROC-ESM_CHEM, Scenario: RCP85

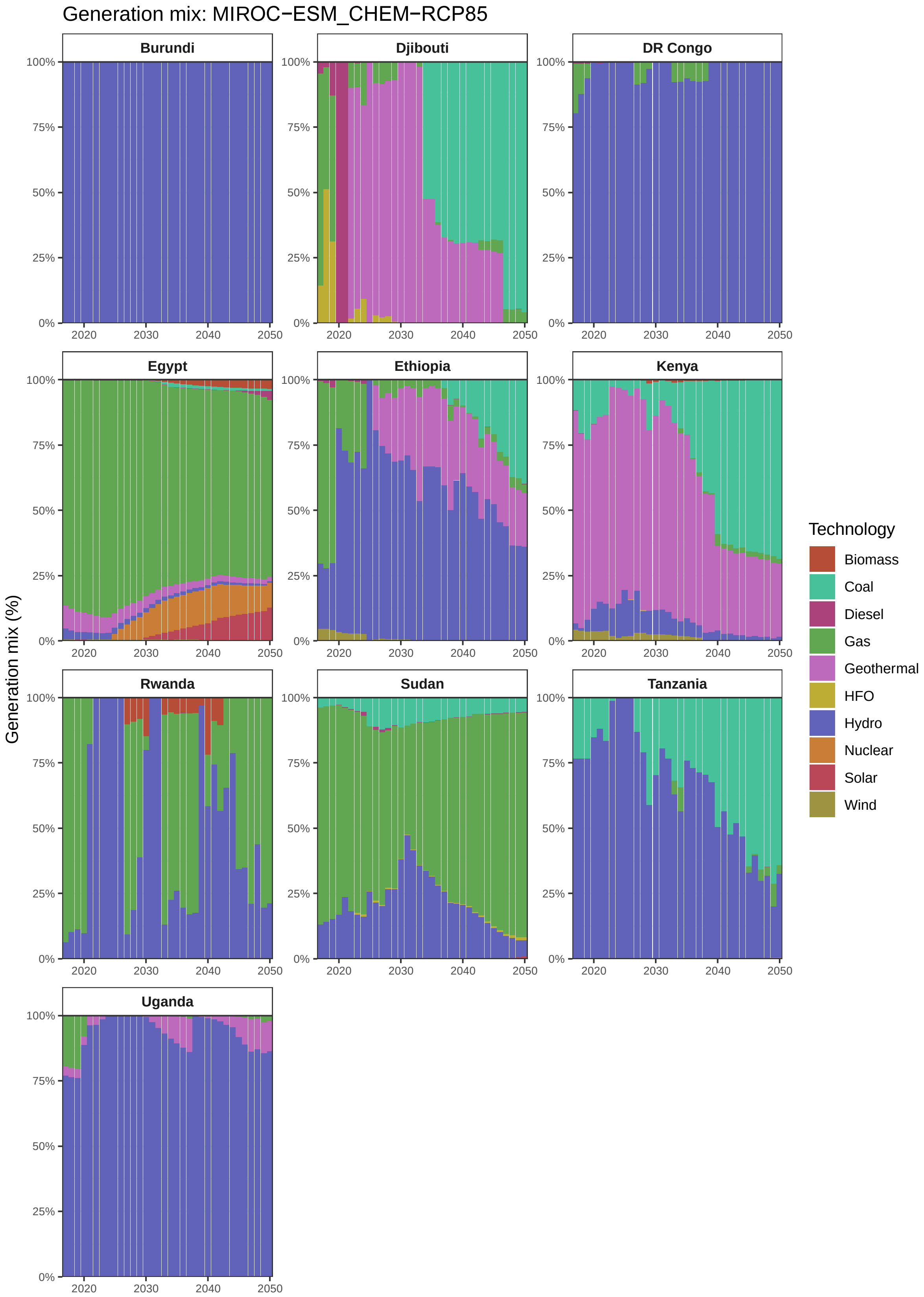

Supplementary Figure 10: Electricity generation mix, GCM: MIROC-ESM_CHEM, Scenario: RCP85

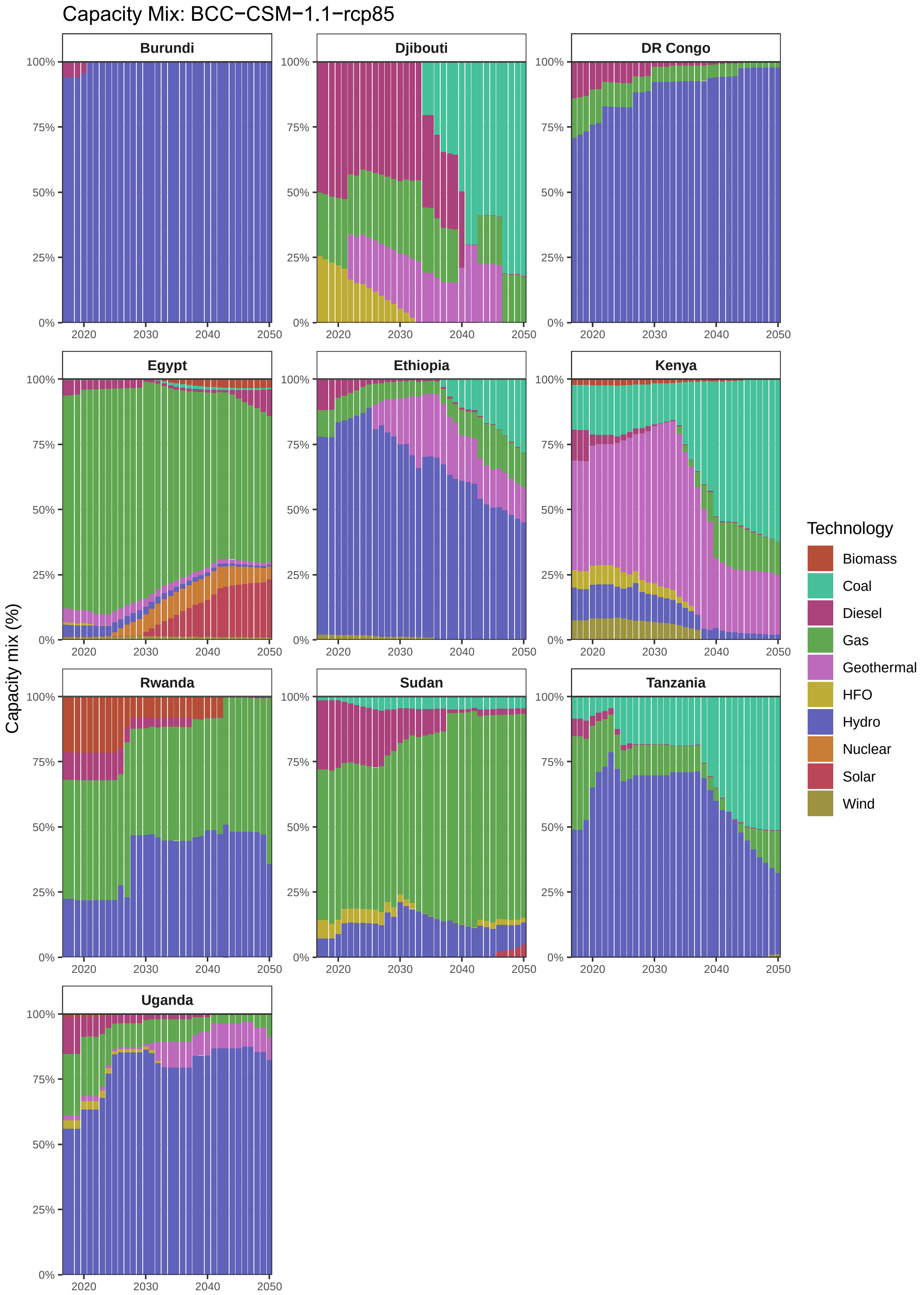

Supplementary Figure 11: Capacity mix. GCM: BCC-CSM-1.1, Scenario: RCP85

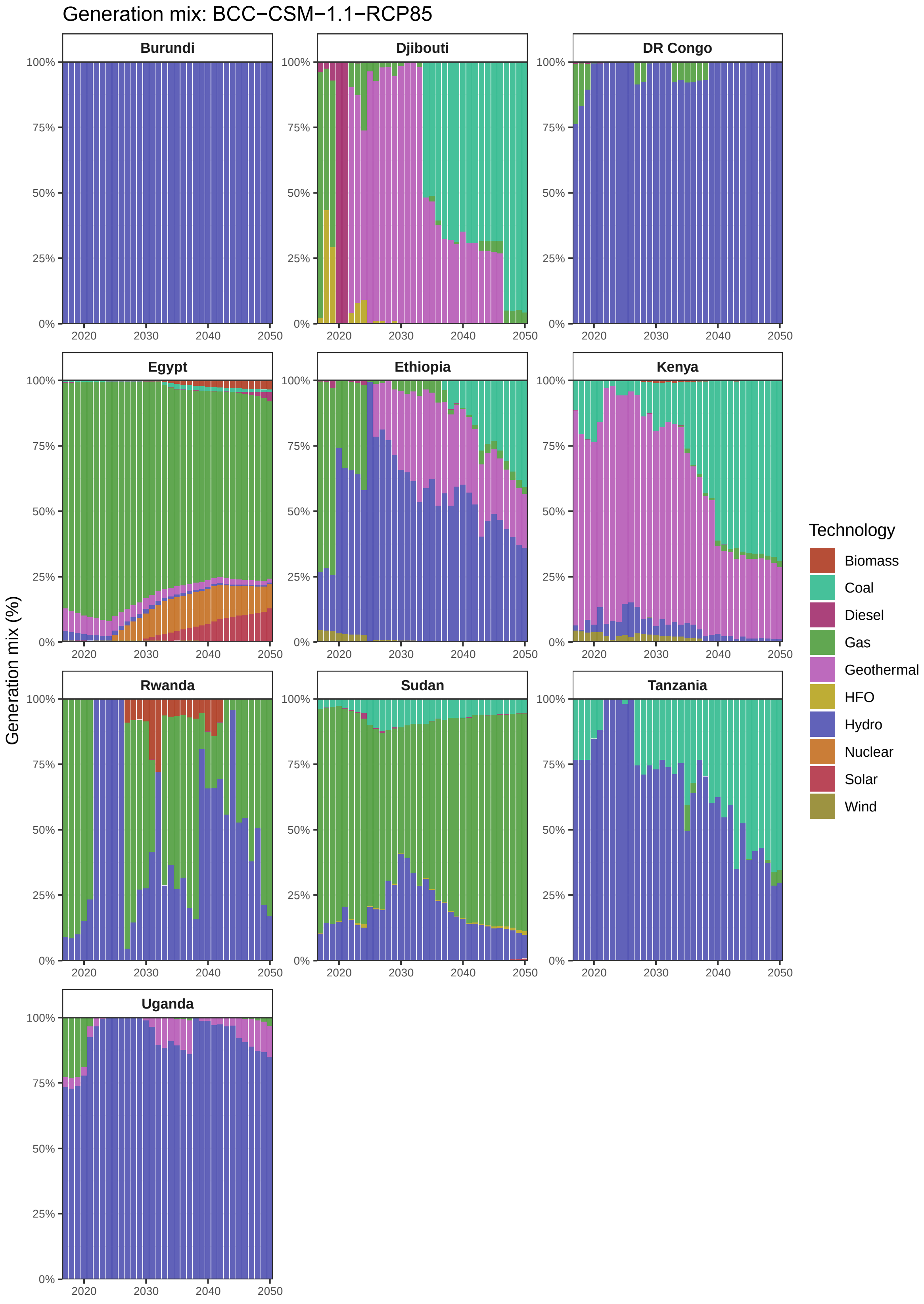

Supplementary Figure 12: Electricity generation mix, GCM: BCC-CSM-1.1, Scenario: RCP85

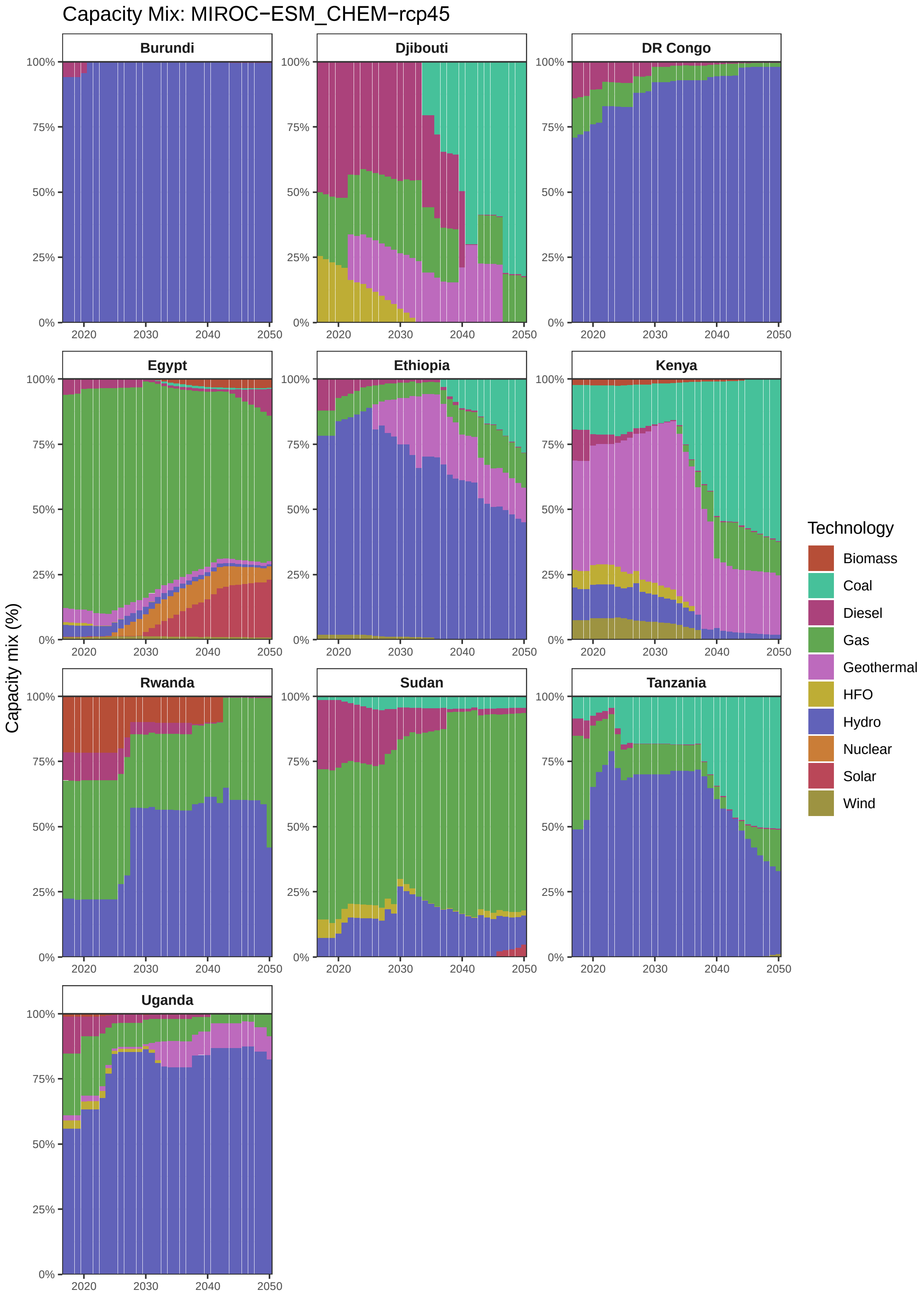

Supplementary Figure 13: Capacity mix, GCM: MIROC-ESM_CHEM, Scenario: RCP45

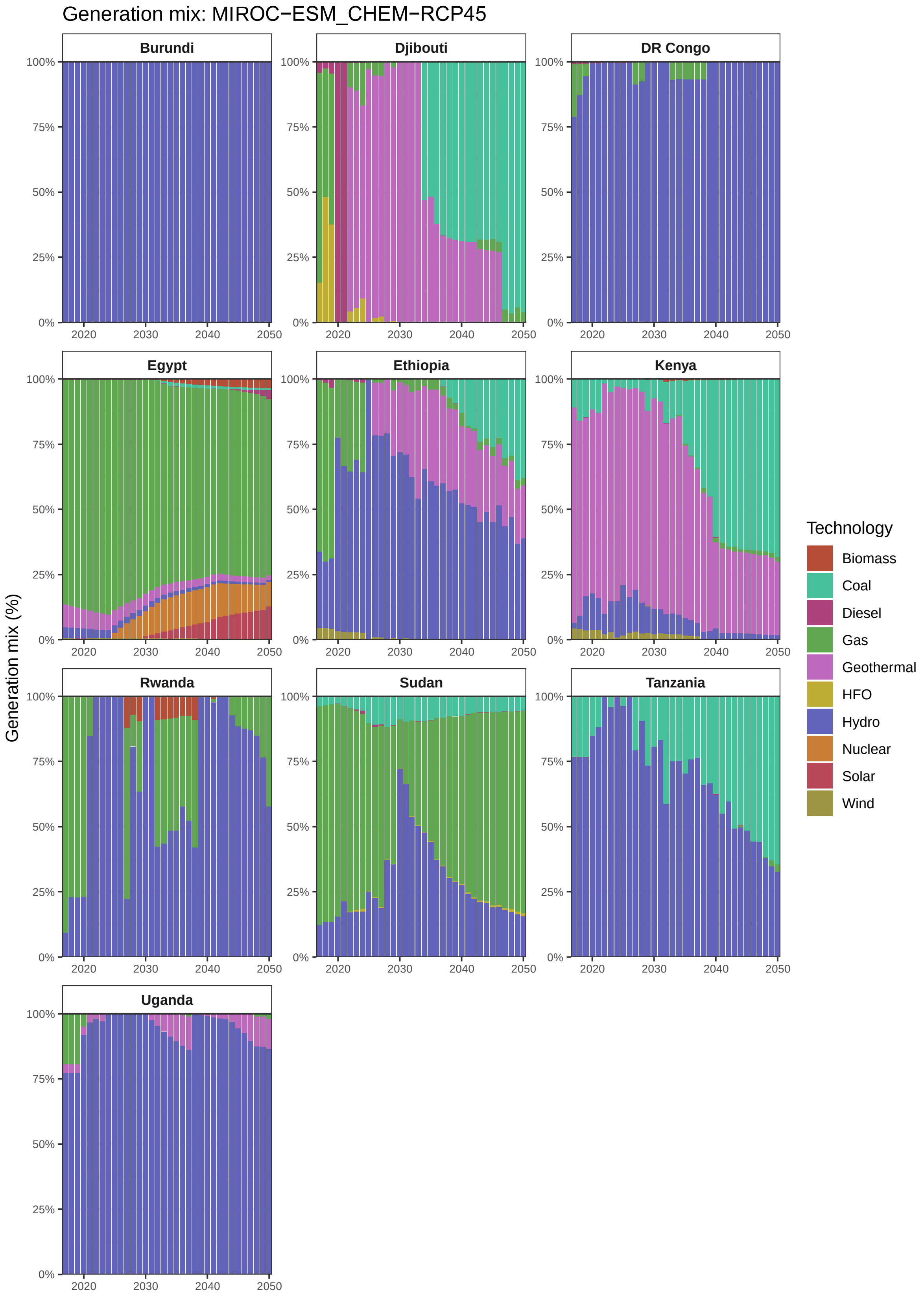

Supplementary Figure 14: Electricity generation mix, GCM: MIROC-ESM_CHEM, Scenario: RCP45

Supplementary Figures 15-16: Perfect foresight adaptation for the wettest and driest climate futures

This section highlights the comparison between the perfect foresight expansion strategies for the wettest and driest climate futures to the baseline strategy. They have been chosen to illustrate the best expansion strategy under the two extremes.

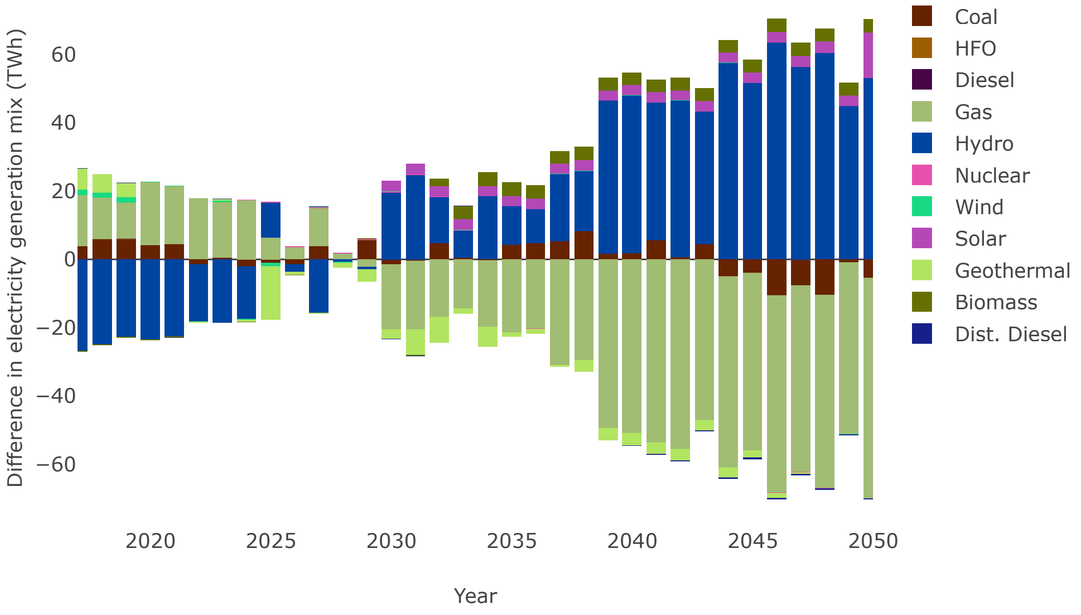

Supplementary Figure 15: Difference in electricity generation mix (TWh) between the PF wettest scenario and the baseline. PF wettest refers to the perfect foresight adaptation for the wettest climate future; baseline refers to the PIDA+ expansion plan for projections reflecting historical climate patterns. The figure also highlights how the electricity system is expected to expand to accommodate climate variability. We can notice that the wettest climate is not uniformly wet; it is cumulatively wetter but has less water availability in the years leading to 2030, represented by the replacement of hydropower generation by gas-based generation. We can also notice that, post-2030, there is increased renewable electricity generation (biomass and solar) under this climate.

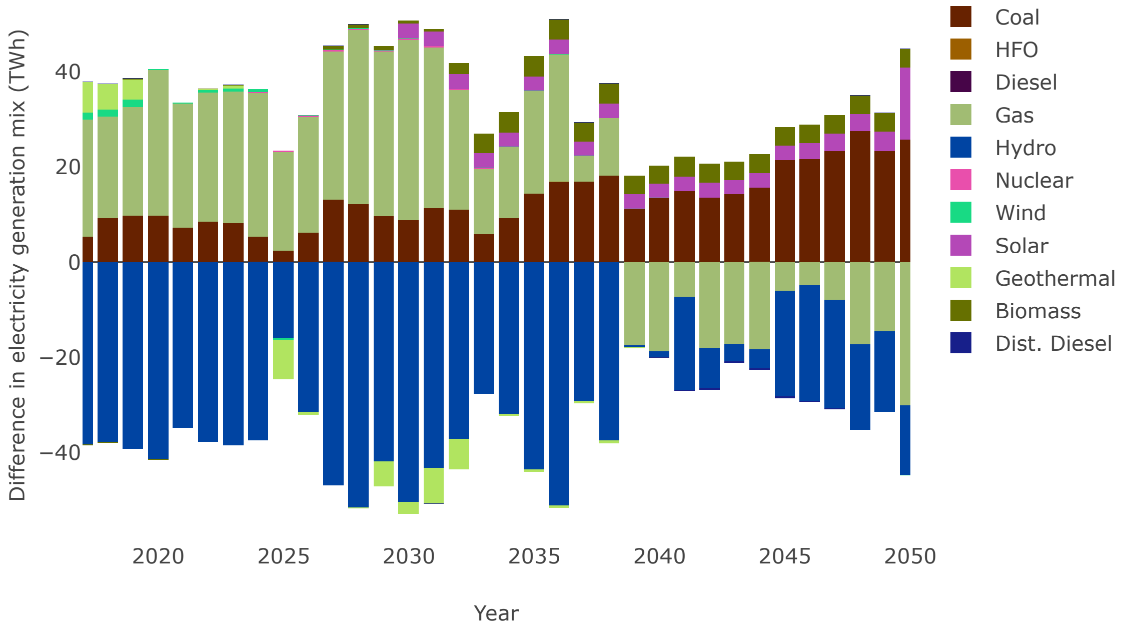

Supplementary Figure 16: Difference in electricity generation mix (TWh) between the PF driest scenario and the baseline. PF driest refers to the perfect foresight adaptation for the driest climate future; baseline refers to the PIDA+ expansion plan for the climate reflecting historical trends. We can notice that the driest climate is predominantly drier compared to the baseline. Natural gas and coal-based generation are expected to contribute to the loss in hydropower generation when compared to the baseline expansion plan.

Supplementary Figures 17-19: Hydropower generation and its impact on the cost of electricity generation

The following figures illustrate the variation in the annualised cost of electricity generation in selected countries along with accumulated hydropower generation. The hydropower generation is plotted to provide an improved understanding of its importance in some countries like Uganda and Tanzania. Whereas, in Egypt the impact is less severe as the share of hydropower is low.

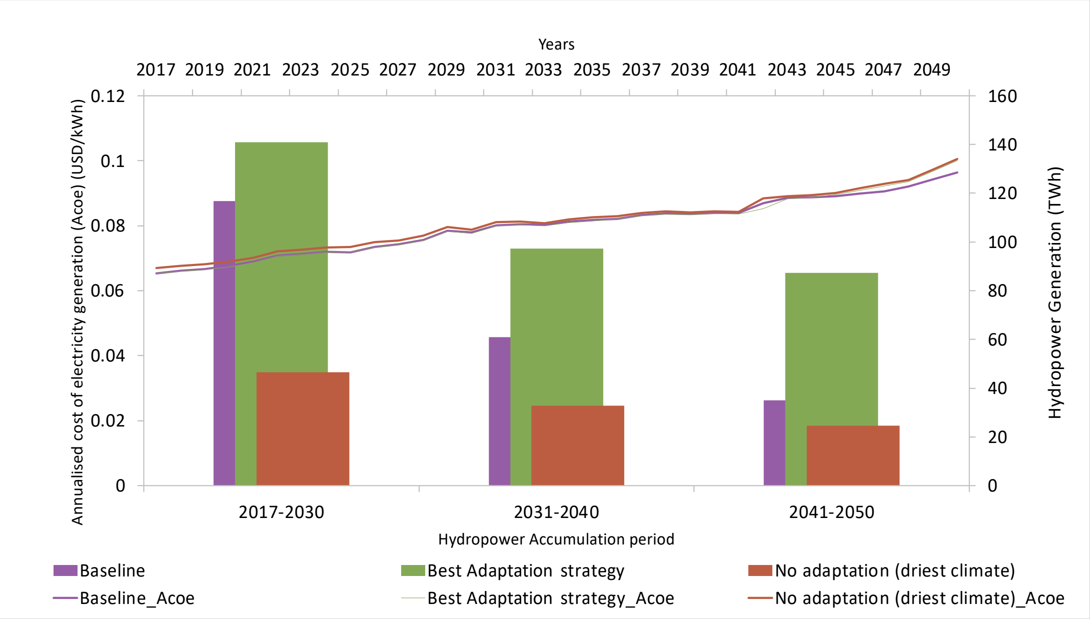

Supplementary Figure 17: The annualised cost of electricity generation (USD/kWh) and corresponding hydropower generation (TWh) in Egypt. The primary X-axis shows the hydropower accumulation period. We can notice that towards the end of the modelling period (2050), all the three climates result in lower hydropower generation. The share of hydropower in Egypt's generation mix is low. Hence, the impact of climatic variation on the cost of electricity generation in low.

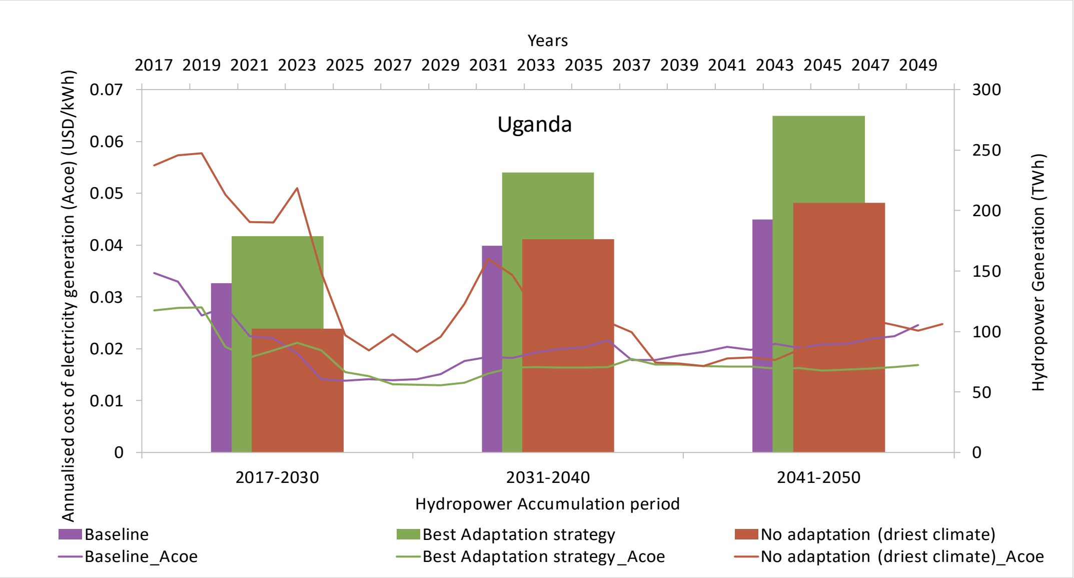

Supplementary Figure 18: The annualised cost of electricity generation (USD/kWh) and corresponding hydropower generation (TWh) in Uganda. The primary X-axis shows the hydropower accumulation period. For Uganda, we can notice the impact of hydro generation on the electricity cost. The share of hydropower in Uganda's generation mix is expected to be high (above 90%). Hence, the impact of climatic variation on the cost of electricity generation is expected to be high. Towards the end of the modelling period, we can notice that the cost of generation drops due to higher shares of hydropower; this also exposes the danger of high fluctuations in terms of climate variability.

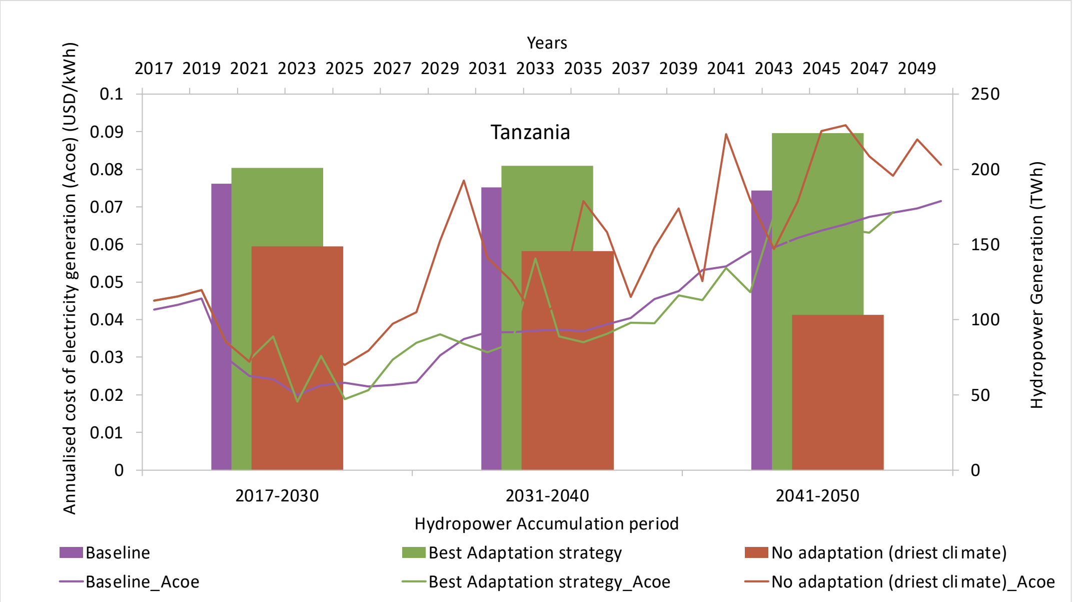

Supplementary Figure 19: The annualised cost of electricity generation (USD/kWh) and corresponding hydropower generation (TWh) in Tanzania. The primary X-axis shows the hydropower accumulation period. For Tanzania, high fluctuation in electricity costs is expected due to climatic variation. Towards the end of the modelling period, the costs are expected to be high due to the increased use of fossil fuels in the generation mix.

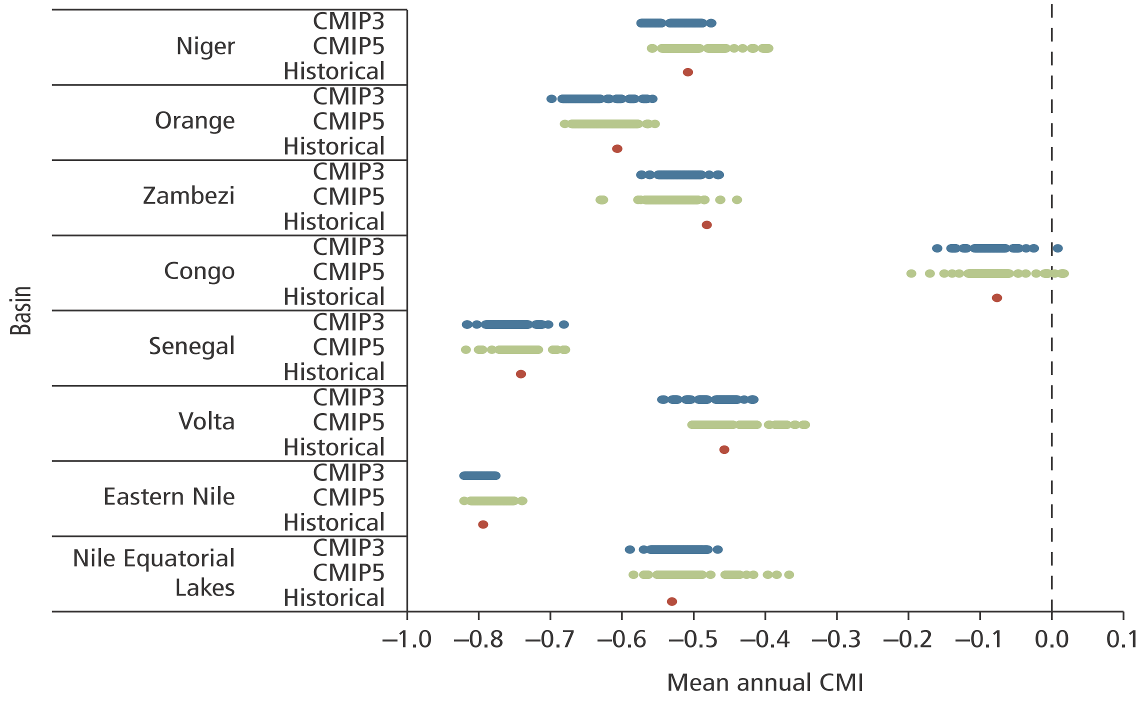

Supplementary Figure 20: Projections of mean annual CMI across Africa's major river basins. The Climate Moisture Index (CMI) is a measure of aridity that combines the effect of rainfall and temperature projections. For example, higher temperatures would increase evaporation. The index values vary between −1 and +1, with lower values representing more arid conditions. A CMI value greater than zero indicates that precipitation rates are greater than potential evapotranspiration rates. CMI is often a good proxy indicator for measures such as river runoff and irrigation demands. The above figure reports CMI values (averaged over the period 2010–50) projected by climate models included in the Intergovernmental Panel on Climate Change (IPCC) fourth and fifth Assessment Reports. In each basin, the red dot denotes the average value of CMI in the historical baseline. Dots to the right of the historical value refer to projections of wetter climate; dots to the left indicate projections of drier climate. CMIP3 corresponds to the IPCC Fourth Assessment General Circulation Model (GCM) results; CMIP5 corresponds to the IPCC Fifth Assessment GCM results (adapted from Cervigni et al. 2016).

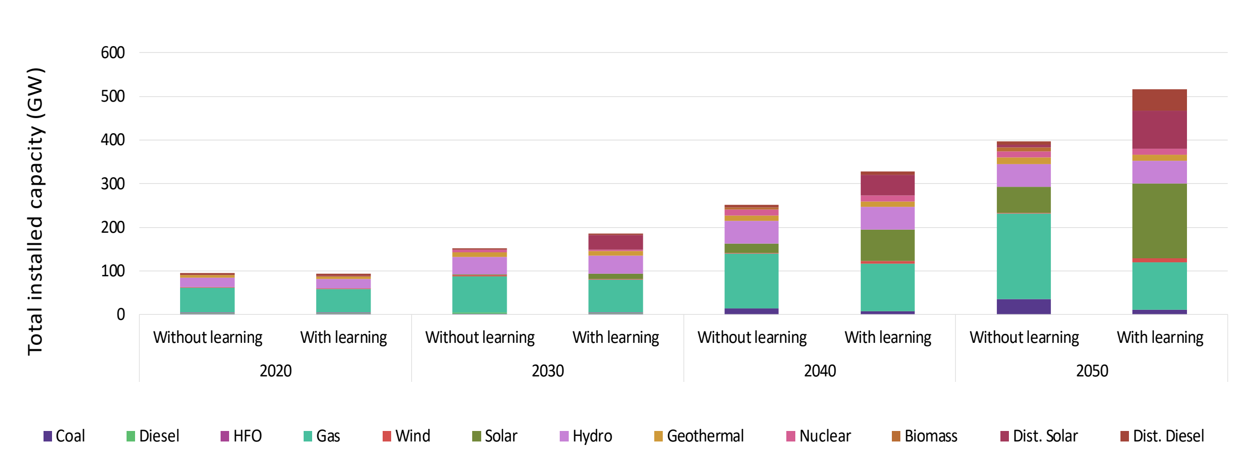

Supplementary Figure 21: Total installed capacity (GW) for the two scenarios, with and without technology learning. With technology learning, the costs of renewable energy technologies are expected to gradually reduce and hence the higher penetration of renewables. The capacity factor of renewable technologies is lower than fossil fuel technologies. Hence, the need for higher generation capacities to meet the same demand, when compared to a scenario without learning.

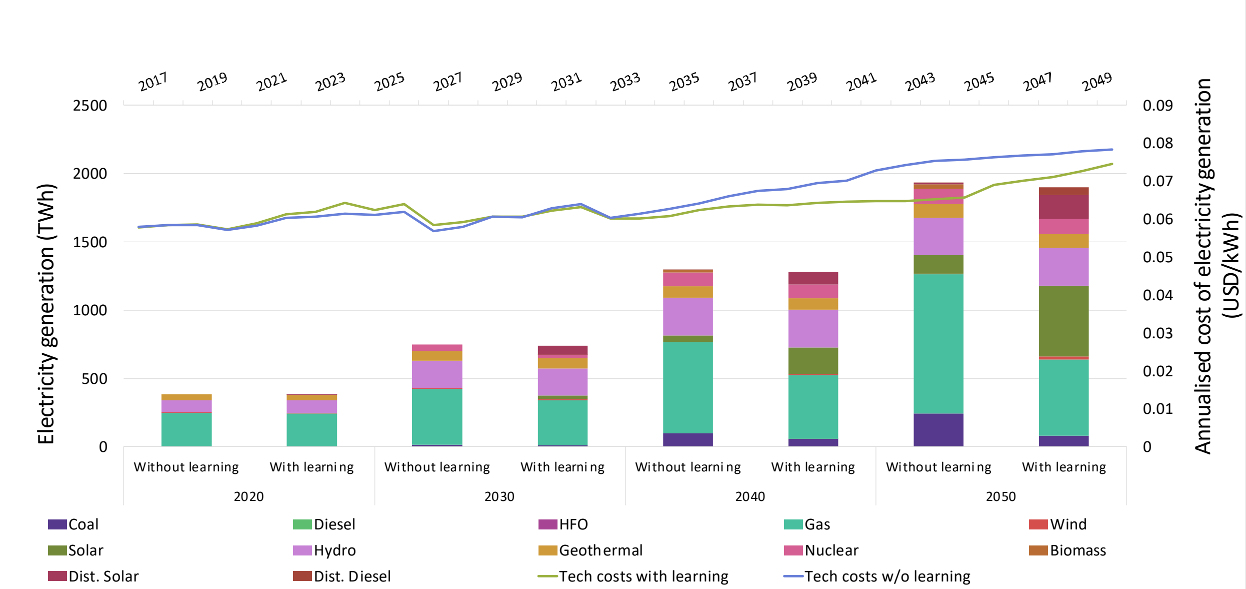

Supplementary Figure 22: Total electricity generation (TWh) in the EAPP for the two scenarios with and without technology learning, along with the annualised cost of electricity generation (USD/kWh). Technology learning contributes to higher renewable penetration in the generation mix of the EAPP. The difference in the cost of electricity generation, towards the end of the modelling period is primarily driven by the reduced usage of fossil fuels (gas) in the generation mix. The hydro generation is practically unaffected.

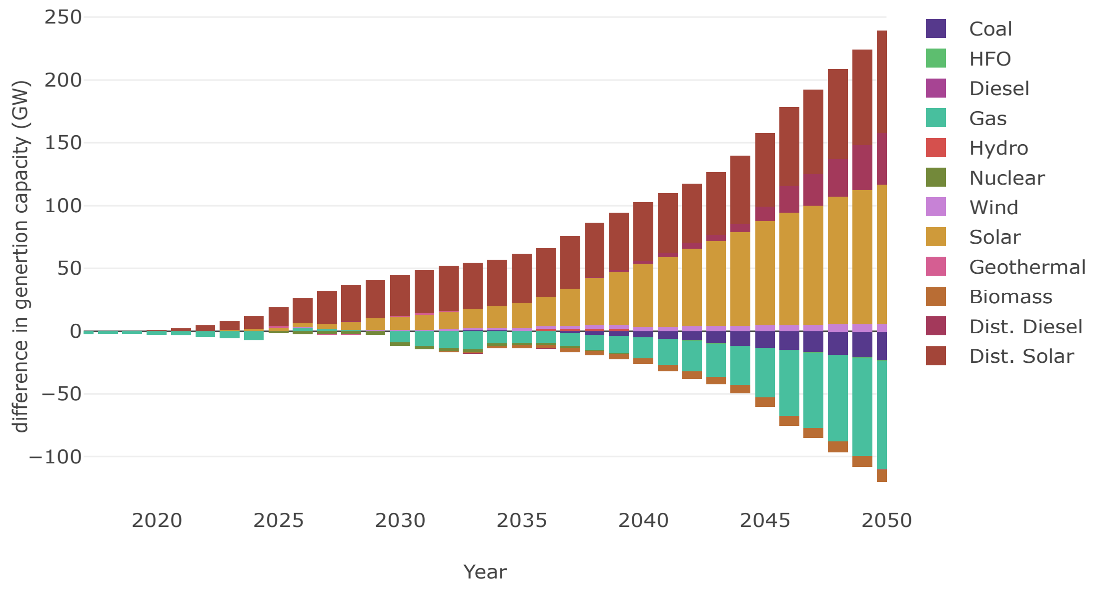

Supplementary Figure 23: Difference in electricity generation capacity (GW) between scenarios with and without renewable technology learning.

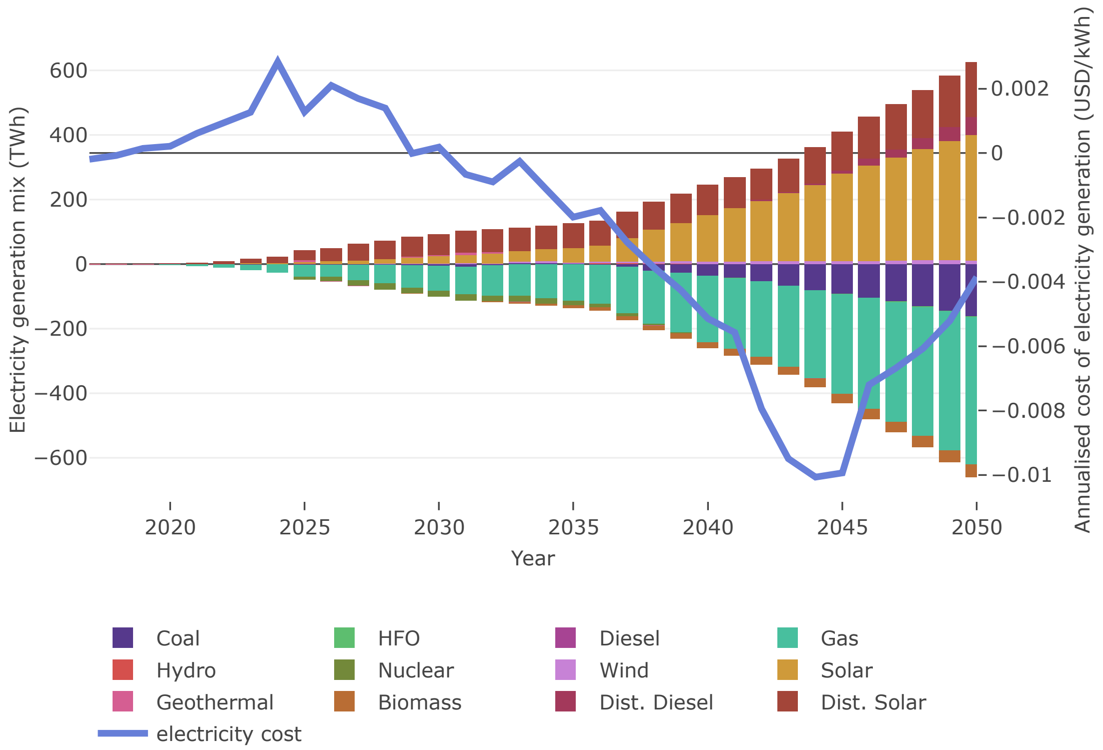

Supplementary Figure 24: Difference in electricity generation (TWh) and cost (USD/kWh) between scenarios with and without renewable technology learning rates. Increased distributed generation also reduces the total primary generation, as there are lower transmission and distribution losses.

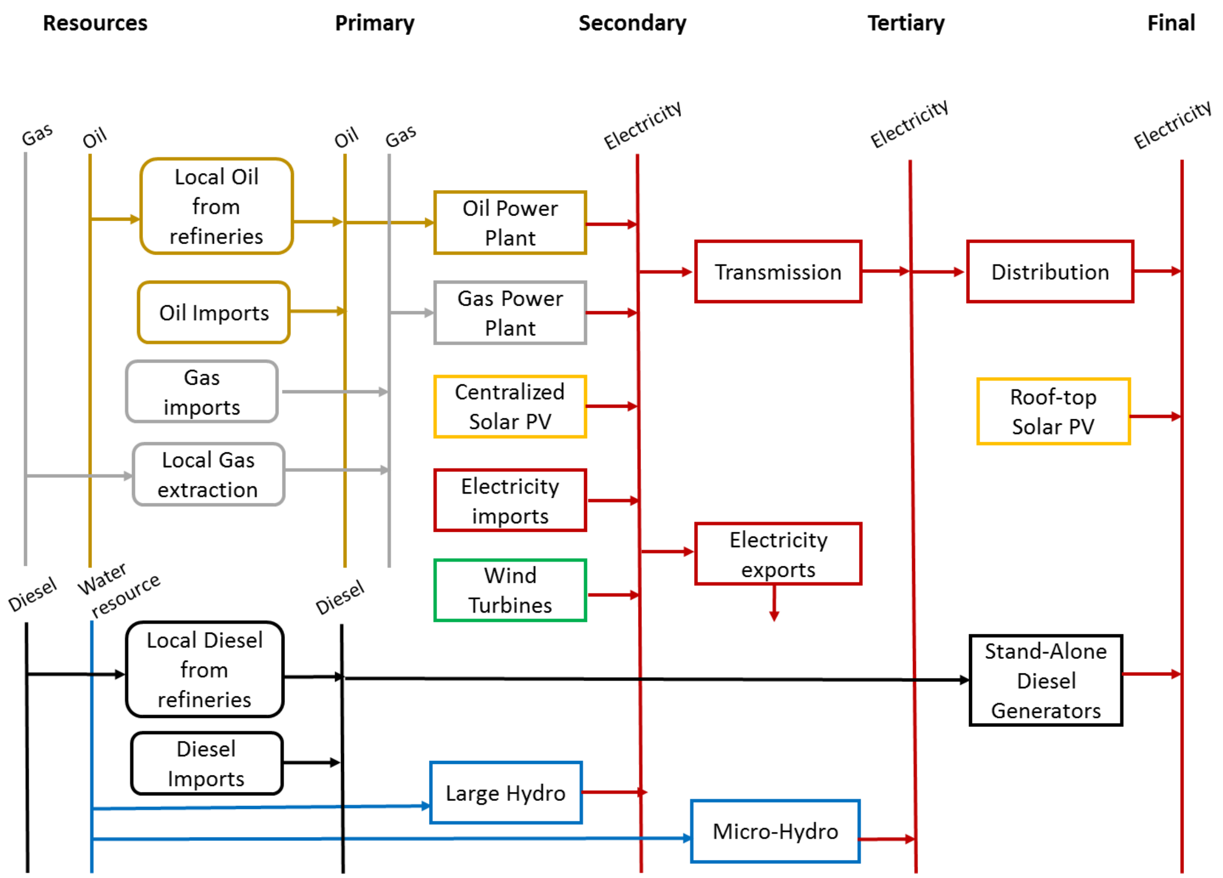

Supplementary Figure 25: EAPP specific reference energy system (RES). The RES is a network representation of all activities required to supply various forms of energy to end-use activities. The illustrated RES is specific to the power sector of the EAPP countries.

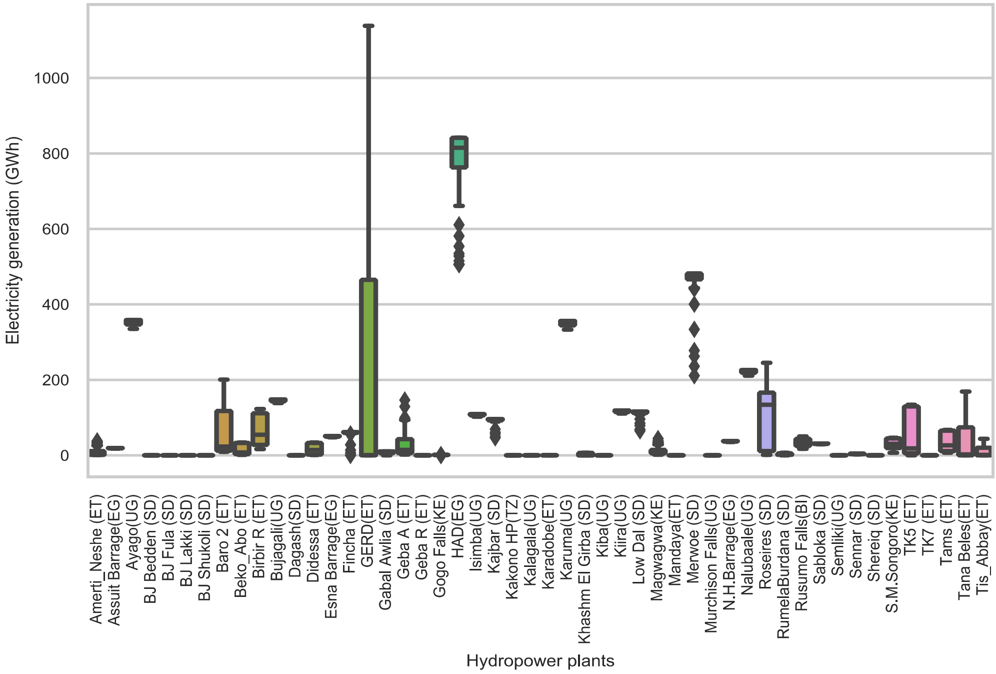

Supplementary Figure 26: Hydropower generation in 2020 from selected hydropower plants. This figure highlights the variation in hydropower generation due to climatic changes (all futures) in the year 2020. The box plots illustrate the variation in generation from plants, both within the same country and across the different countries in the power pool.

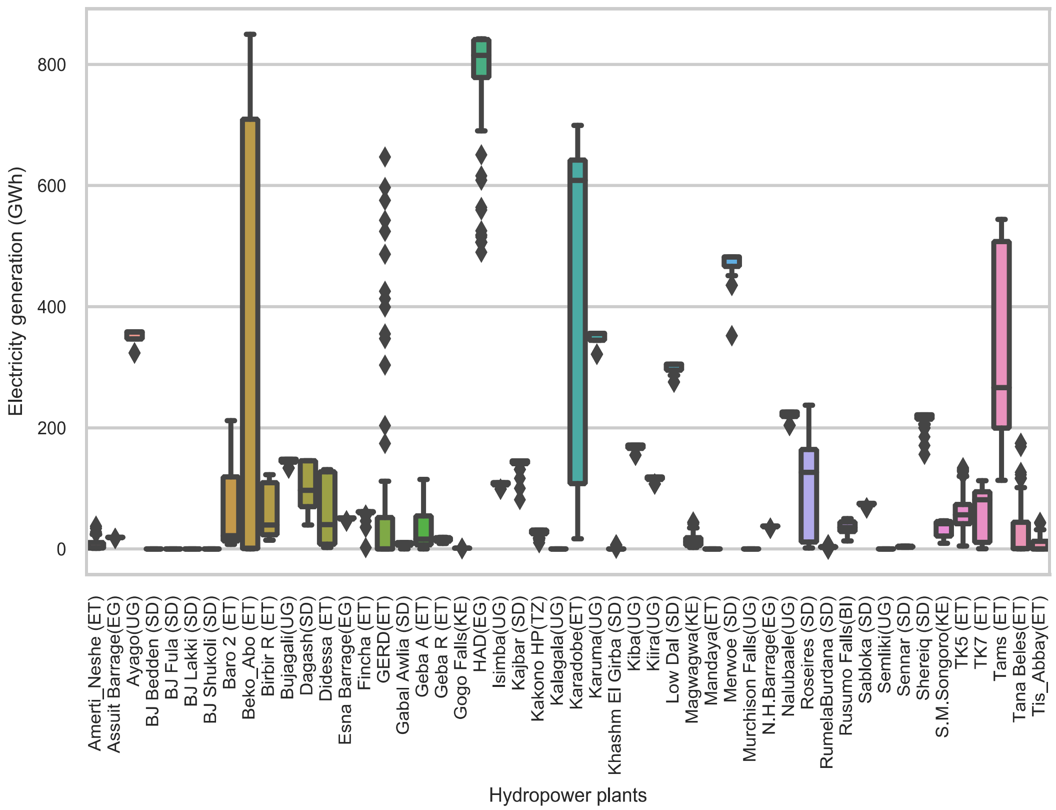

Supplementary Figure 27: Hydropower generation in 2030 from selected hydropower plants, across the different climate futures. This figure highlights the variation in hydropower generation due to climatic changes in the year 2030. The box plot illustrates the variation in generation from plants, both within the same country and across the different countries in the power pool

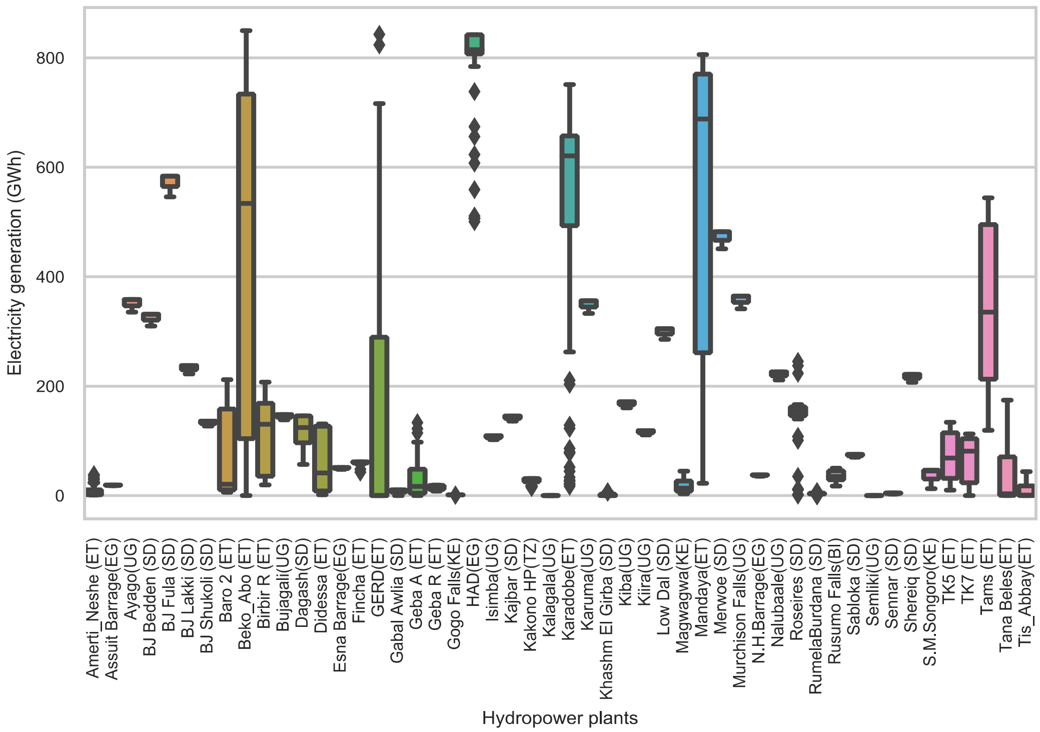

Supplementary Figure 28: Hydropower generation in 2040 from selected hydropower plants, across the different climate futures. This figure highlights the variation in hydropower generation due to climatic changes in the year 2040. The box plot illustrates the variation in generation from plants, both within the same country and across the different countries in the power pool.

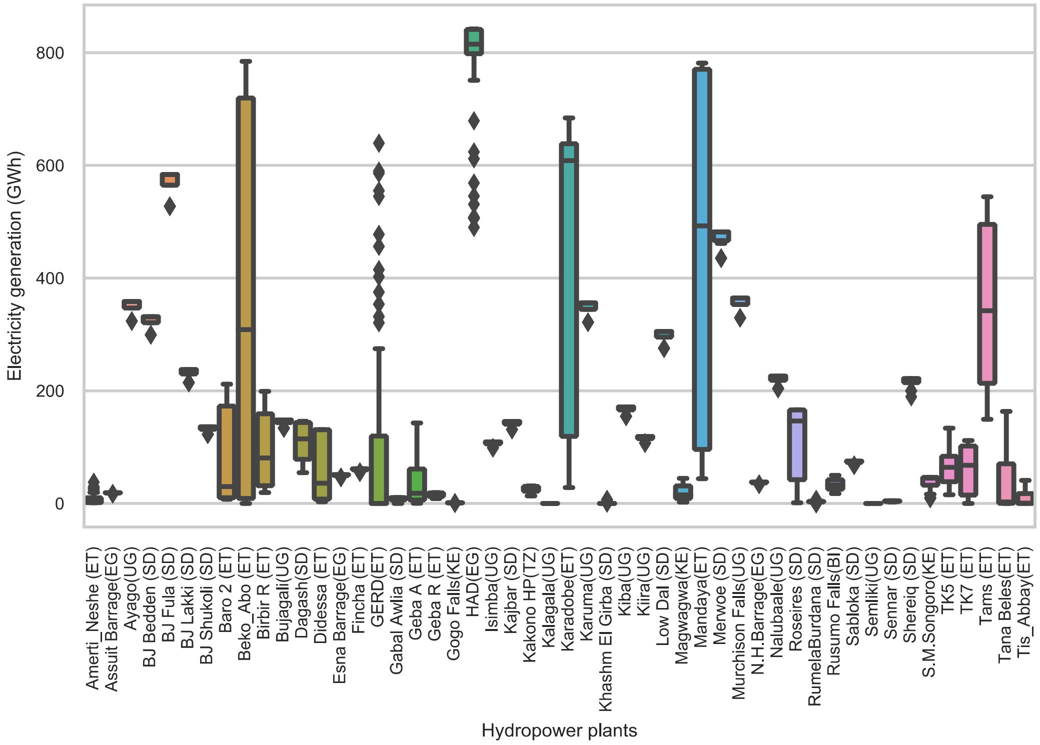

Supplementary Figure 29: Hydropower generation in 2050 from selected hydropower plants, across the different climate futures. This figure highlights the variation in hydropower generation due to climatic changes in the year 2050. The box plot illustrates the variation in generation from plants, both within the same country and across the different countries in the power pool

Supplementary Table 1: Techno-Economic Data for generic power power plants in the EAPP [48]

Power Plant (Technologies)	Capital Cost ($/kW)-10% IDC	Capital Cost ($/kW)-5% IDC	Variable O&M Cost (USD/GJ)	Lifetime (Years)	Construction time (Years)
Biomass	3660	3039	5.56	30	4
Coal	3519	2921	3.96	35	4
Diesel 100 kW (Industrial)	659	659	15.38	20	0
Diesel 1kW (Rural)	692	692	9.23	10	0
Diesel 1kW (Urban)	692	692	9.23	10	0
Diesel (Centralized)	1177	1124	4.72	30	1
Geothermal	5856	4862	1.39	25	4
Heavy Fuel Oil (HFO)	1634	1488	4.17	25	2
Gas Turbine (Combined cycle)	1423	1238	0.80	30	3
Gas turbine (Other cycles)	730	665	5.53	25	2
Nuclear	10778	7429	3.87	60	8
CSP	4392	3647	6.20	25	4
CSP with Storage	10249	8509	4.56	25	4
CSP with Gas Co-firing	2033	1687	4.56	25	4
Solar PV Utility	2200	2100	5.58	25	1
PV Rural Rooftop	2100	2100	4.16	20	<1
PV Rural rooftop 1hr storage	4258	4258	4.16	20	<1
PV Rural rooftop 2hr storage	6275	6275	4.76	20	<1
PV Urban Rooftop	2100	2100	4.16	20	<1
PV Urban rooftop 1hr storage	4258	4258	4.76	20	<1
PV Urban rooftop 2hr storage	6275	6275	5.29	20	<1
Wind 25% Capacity Factor	2861.65	2607	3.97	25	2
Wind 30% Capacity Factor	2420	2205	3.97	25	2
*Large Hydro**	3221	2553	1.66	50	5
*Micro Hydro**	4800	4410	1.51	30	2

* All the site-specific hydropower plants have specific costs. This generic value is used only when it is not possible to estimate the cost of an investment in the future

Supplementary Table 2: Set of climate futures chosen for this assessment. A set of futures (combination of GCMs and emission scenarios) are chosen from both the CMIP3 and CMIP5 suite of models. The EAPP specific precipitation and temperature projections for the chosen climates are used in the water model for deriving water availability at different hydropower plants.

Climate numbering used for the study	GCM	Emission scenario	Vintage
**124**	BCC-CSM 1.1	RCP 8.5	CMIP5
**80**	GISS-E2-H	RCP 4.5	CMIP5
**35**	IPSL-CM4	A2	CMIP3
**39**	MIROC3_2_medres	A1B	CMIP3
**137**	MIROC-ESM-CHEM	RCP 4.5	CMIP5
**90**	MIROC-ESM_CHEM	RCP 8.5	CMIP5

Supplementary Table 3: Net present value (NPV) of consumer electricity expenditure for all climate strategies in billion $USD_{2010.}$ The NPVs are calculated for the different climates and the respective strategies using the total consumer expenditures for electricity as a metric.

	Climate 137	***Climate 124***	***Climate 90***	***Climate 80***	***Climate 39***	***Climate 35***	***Climate 0***
Strategy 137	943.61	803.78	883.62	603.42	868.24	759.09	831.06
Strategy 124	934.24	800.89	888.81	584.45	870.74	751.17	825.56
Strategy 90	911.77	782.9	871.86	567.95	863.16	732.61	800.89
Strategy 80	933.67	797.16	882.18	587.36	865.53	746.05	827.34
Strategy 39	918.85	784.95	872.27	573.55	866.81	736	802.73
Strategy 35	927.34	799.76	886.19	584.69	879.46	749.4	823.18
Strategy 0	896.39	790.28	853.47	592.2	844.67	740.59	813.61

Supplementary Table 4: Financial regret for each climate strategy across different climates in billion $USD_{2010.}$ The difference in the NPV of the assessed strategy to the best performing strategy for that climate is referred to as the regret.

	Climate 137	***Climate 124***	***Climate 90***	***Climate 80***	***Climate 39***	***Climate 35***	***Climate 0***
Strategy 137	0	0	5.19	0	11.22	0	0
Strategy 124	9.37	2.89	0	18.97	8.72	7.92	5.5
Strategy 90	31.84	20.88	16.95	35.47	16.3	26.48	30.17
Strategy 80	9.94	6.62	6.63	16.06	13.93	13.04	3.72
Strategy 39	24.76	18.83	16.54	29.87	12.65	23.09	28.33
Strategy 35	16.27	4.02	2.62	18.73	0	9.69	7.88
Strategy 0	47.22	13.5	35.34	11.22	34.79	18.5	17.45

Supplementary tables 5-15: Data used for the EAPP model

Supplementary Table 5: Population projections for the EAPP countries in millions

	2015	2020	2025	2030	2035	2040	2045	2050
Ethiopia	98.9	111.5	124.5	137.7	150.7	163.6	175.9	187.6
Egypt	84.7	91.1	97.0	102.6	107.9	113.0	117.7	121.8
Tanzania	52.3	60.4	69.3	79.4	90.5	102.7	115.7	129.4
Kenya	46.7	52.9	59.4	66.3	73.7	81.4	89.2	97.2
Sudan	39.6	44.5	49.7	55.1	60.6	66.2	71.7	77.1
Uganda	40.1	47.1	54.8	63.4	72.7	82.7	93.2	104.1
Rwanda	12.4	14.1	15.9	17.8	19.7	21.6	23.5	25.4
Burundi	10.8	12.6	14.4	16.4	18.6	21.1	23.8	26.7
Djibouti	0.9	1.0	1.0	1.1	1.1	1.2	1.2	1.2

Supplementary Table 6: Electricity demand in the EAPP countries (TWh)

	2015	2020	2025	2030	2035	2040	2045	2050
BURUNDI	*0.20*	*0.38*	*0.63*	*0.97*	*1.39*	*1.85*	*2.42*	*3.07*
DJIBOUTI	*0.73*	*0.91*	*1.00*	*1.10*	*1.21*	*1.33*	*1.46*	*1.60*
EGYPT	*185.40*	*246.65*	*324.72*	*423.91*	*547.47*	*691.26*	*854.08*	*1037.31*
ETHIOPIA	*8.90*	*14.51*	*21.56*	*31.79*	*46.91*	*65.15*	*86.11*	*110.27*
KENYA	*13.75*	*20.77*	*30.76*	*45.27*	*65.25*	*89.26*	*116.96*	*148.80*
RWANDA	*0.56*	*0.96*	*1.52*	*2.23*	*3.09*	*4.14*	*5.33*	*6.66*
SUDAN	*12.19*	*21.42*	*34.92*	*54.29*	*79.95*	*110.72*	*146.64*	*187.91*
TANZANIA	*7.50*	*11.35*	*15.27*	*20.57*	*27.97*	*37.67*	*51.51*	*69.73*
UGANDA	*3.81*	*5.12*	*6.85*	*8.92*	*11.18*	*13.75*	*16.68*	*19.92*

Supplementary Table 7: Calculated EAPP year split definition

	Jan	Feb	Mar	Apr	May	Jun	Jul	Aug	Sep	Oct	Nov	Dec
Weekday, Low demand	1.95%	1.76%	1.95%	1.88%	1.95%	1.88%	1.95%	1.95%	1.88%	1.95%	1.88%	1.95%
Weekday, High Demand	4.60%	4.16%	4.60%	4.46%	4.60%	4.46%	4.60%	4.60%	4.46%	4.60%	4.46%	4.60%
Weekend, High demand	1.42%	1.28%	1.42%	1.37%	1.42%	1.37%	1.42%	1.42%	1.37%	1.42%	1.37%	1.42%
Weekend, Low demand	0.53%	0.48%	0.53%	0.51%	0.53%	0.51%	0.53%	0.53%	0.51%	0.53%	0.51%	0.53%

Supplementary Table 8: Calculated share of the demand to be met in each time slice

	Jan	Feb	Mar	Apr	May	Jun	Jul	Aug	Sep	Oct	Nov	Dec
Weekday, Low demand	1.54%	1.39%	1.54%	1.46%	1.50%	1.43%	1.47%	1.44%	1.40%	1.44%	1.40%	1.44%
Weekday, High Demand	5.00%	4.51%	4.99%	4.73%	4.89%	4.64%	4.79%	4.69%	4.54%	4.69%	4.54%	4.69%
Weekend, High demand	1.86%	1.68%	1.86%	1.76%	1.82%	1.73%	1.78%	1.75%	1.69%	1.75%	1.69%	1.75%
Weekend, Low demand	0.42%	0.38%	0.42%	0.40%	0.41%	0.39%	0.40%	0.39%	0.38%	0.39%	0.38%	0.39%

Supplementary Table 9: Identified fossil reserves in the EAPP countries (TWh)

Country	**Coal***	**Crude Oil ****	**Natural Gas**
Burundi	0.00	0.00	0.00
Djibouti	0.00	0.00	0.00
Egypt	99.97	7811.90	23100.3
Ethiopia	0.00	0.76	263.32
Kenya	0.00	0.00	0.00
Rwanda	0.00	0.00	598.45
Sudan	0.00	8877.16	897.68
Tanzania	1249.65	0.00	68.82
Uganda	0.00	4438.58	149.61

*2008 data, **2011 data

Source: [49]

Supplementary Table 10: Fossil fuel cost projections USD/GJ [50]

	Diesel	Heavy Fuel Oil	Coal	Natural Gas
2013	15.3	13.6	3.6	5.8
2014	16	14.3	3.3	5.8
2015	16.4	14.7	3.4	5.9
2016	17.2	15.1	3.3	6
2017	17.8	15.6	3.4	6.1
2018	18.4	16	3.3	6.1
2019	18.8	16.2	3.3	6.2
2020	19.1	16.3	3.3	6.3
2021	19.3	16.5	3.3	6.4
2022	19.4	16.7	3.3	6.6
2023	19.7	16.9	3.3	6.6
2024	19.9	17.1	3.3	6.6
2025	20.2	17.3	3.3	6.6
2026	20.4	17.4	3.3	6.8
2027	20.6	17.7	3.3	6.9
2028	20.9	17.9	3.3	7.1
2029	21.3	18.2	3.3	7.4
2030	21.5	18.5	3.4	7.6
2031	21.9	18.8	3.4	8
2032	22.2	19.1	3.4	8.1
2033	22.6	19.3	3.4	8.1
2034	22.9	19.6	3.4	8.3
2035	23.3	20	3.5	8.4
2036	23.8	20.3	3.5	8.5
2037	24.2	20.6	3.5	8.7
2038	24.6	21	3.5	8.8
2039	24.6	21	3.5	8.8
2040	24.6	21	3.5	8.8
2041	24.6	21	3.5	8.8
2042	24.6	21	3.5	8.8
2043	24.6	21	3.5	8.8
2044	24.6	21	3.5	8.8
2045	24.6	21	3.5	8.8
2046	24.6	21	3.5	8.8
2047	24.6	21	3.5	8.8
2048	24.6	21	3.5	8.8
2049	24.6	21	3.5	8.8
2050	24.6	21	3.5	8.8

Supplementary Table 11: Renewable Energy potential per country

	[TWh per year]			
	CSP	PV	Wind	
			20 CF	30 CF
Burundi	785	888	0	0
Djibouti	851	946	934	226
Egypt	26604	32218	36601	6757
Ethiopia	22959	27154	14838	4983
Kenya	15399	23045	22476	6185
Rwanda	788	892	0	0
Sudan	77422	87817	61661	12784
Tanzania	31842	38804	18455	3084
Uganda	8581	9470	815	125

Supplementary Table 12: Electricity generation capacity in the EAPP (GW)

**Country**	**Hydro (MW)**		**Coal (MW)**		**Nuclear (MW)**		**Renewables (Non Hydro) (MW)**		**Natural gas (MW)**		**Liquid Fossil Fuels (MW)**	
	Historic	*Planned*	*Historic*	*Planned*	*Historic*	*Planned*	*Historic*	*Planned*	*Historic*	*Planned*	*Historic*	*Planned*
Burundi	36	90	0	0	0	0	0	0	0	0	6	10
Djibouti	0	0	0	0	0	0	0	52	0	0	172	3
Ethiopia	2138	15872	0	0	0	0	231	411	0	0	108	0
Kenya	817	144	0	1131	0	0	589	1956	0	700	823	232
Rwanda	80	111	0	0	0	0	9	20	29	125	43	0
Sudan	1592	695	0	600	0	0	0	0	0	0	1025	0
Tanzania	571	2936	27	1325	0	0	0	460	649	1476	498	70
Uganda	706	1553	0	0	0	0	0	170	0	0	225	287
Egypt	2842	688	0	6270	0	4800	634	344	25881	30255	1001	0

Supplementary Table 13: Site specific Hydropower plant parameters

Power Plant Name	WEAP Proxy	River Basin	Capacity (MW)	Capital Cost ($/kW)	Fixed Cost ($/kW)	Variable Cost ($/GJ)	Status	Earliest on
			Burundi					
Consolidated Historic	Rusumo Falls	Nile	30.1	0	21	0.32	Historic	
Kabu 16	Rusumo Falls	Nile	20	2943	3.826	0.06	COM	2015
Mphanda	Rusumo Falls	Nile	10	6548	4.108	0.06	COM	2016
Siguvyayae	Rusumo Falls	Nile	90	4869	3.824	0.06	PLN	2016
Rusumo	Rusumo Falls	Nile	20	0	21	0.32	PLN	2017
Ruzizi III	Rusumo Falls	Nile	48.3	2553	21	0.32	PLN	2018
Ruzizi IV	Rusumo Falls	Nile	95.7	2553	21	0.32	PLN	2019
Mule 34	Rusumo Falls	Nile	17	3070	21	0.32	PLN	2016
Jiji 3	Rusumo Falls	Nile	16	4179	21	0.32	PLN	2016
Kaganuzi A	Rusumo Falls	Nile	34	2296	21	0.32	PLN	2016
Kaganuzi Complex	Rusumo Falls	Nile	39	5357	21	0.32	PLN	2016
Ruzizi II (Historic)	Rusumo Falls	Nile	12	2553	21	0.32	Historic	
			Egypt					
High Aswan Dam	-	Nile	2100	0	21	0.32	Historic	
Esna	-	Nile	85.8	0	21	0.32	Historic	
Nagaa Mamadi	-	Nile	640	0	21	0.32	Historic	
Aswan	HAD	Nile	592	0	21	0.32	Historic	
Gabal Galala	-	Nile	650	2552	21	0.32	PLN	2018
Asyut	-	Nile	32	2552	21	0.32	PLN	2017
Zefta	Assuit Barrage	Nile	5.5	2552	21	0.32	PLN	2018
Faiyun	Assuit Barrage	Nile	0.8	2552	8.5713	0.32	Historic	
			Ethiopia					
Gibe II	Geba A Dam	Nile	420	0	21	0.32	Historic	
Tana Beles	-	Nile	460	2553	21	0.32	COM	
Tekeze I	-	Nile	300	0	21	0.32	Historic	
Gibe III	Geba A Dam	Nile	1870	1148	4.249	0.06	COM	2013
Gibe IV	Geba A Dam	Nile	1468	1899	4.137	0.06	COM	2015
Halele Worabesa	Geba A Dam	Nile	422	1438	3.891	0.06	COM	2014
Chemoga Yeda	Geba A Dam	Nile	280	1722	3.942	0.06	COM	2016
Geba I	-	Nile	214.5	1680	3.959	0.06	PLN	2025
Genale 3D	Geba A Dam	Nile	258	1410	3.973	0.06	COM	2015
Baro 1 and 2 + Genji	-	Nile	900	4308	3.928	0.06	PLN	2025
Mandaya	-	Nile	2200	1495	3.775	0.06	COM	2035
Border	GERD	Nile	1200	1706	3.887	0.06	PLN	2019
GIbe V	Geba A Dam	Nile	662	1672	4.332	0.07	PLN	2019
Beko Abo	-	Nile	935	1820	3.907	0.06	PLN	2025
Karadobi	-	Nile	1600	2173	3.852	0.06	PLN	2025
Genale 6D	Geba A Dam	Nile	246	1863	3.69	0.06	COM	2016
Gojeb	Geba A Dam	Nile	150	2251	4.2	0.06	COM	2016
Tekeze II TK7	-	Nile	450	5071	4.125	0.06	PLN	2025
Aleltu East	Geba A Dam	Nile	186	2906	3.974	0.06	PLN	2018
Aleltu West	Geba A Dam	Nile	265	2612	4.146	0.06	PLN	2019
Awash 4	Fincha Dam	Nile	38	1559	4.042	0.06	COM	2016
Amerti neshe	-	Nile	97	0	21	0.32	Historic	
Fincha	-	Nile	128	0	21	0.32	Historic	
Tis abbay (1&2)	-	Nile	85.2	0	21	0.32	Historic	

Awash (1,2,3)	Fincha Dam	Nile	107	0	21	0.32	Historic		
Malka Wajana	Geba A Dam	Nile	153	0	21	0.32	Historic		
Gilgel Gibe 1	Geba A Dam	Nile	192	0	21	0.32	Historic		
Lower Didessa	-	Nile	550	1463	21	0.32	PLN	2025	
Grand Renaissance	-	Nile	6000	800	21	0.32	COM	2017	
Birbir R	-	Nile	465	3442	21	0.32	PLN	2035	
Tams	-	Nile	1060	7406	21	0.32	PLN	2020	
Geba 2	-	Nile	157	957	3.959	0.06	PLN	2025	
				Kenya					
Gogo falls	-	Nile	2	0	21	0.32	Historic		
Sondo-Miriu Songoro	-	Nile	81.2	0	21	0.32	Historic		
Kambaru	Magwagwa	Nile	94	0	21	0.32	Historic		
Gitaru	Magwagwa	Nile	225	0	21	0.32	Historic		
Kindaruma	Magwagwa	Nile	40	0	21	0.32	Historic		
Masinga	Magwagwa	Nile	40	0	21	0.32	Historic		
Kiambere	Magwagwa	Nile	164	0	21	0.32	Historic		
Turkwell	Magwagwa	Nile	106	0	21	0.32	Historic		
Consolidated (Tana,Wanji,Misc)	Magwagwa	Nile	37	0	21	0.32	Historic		
Magwagwa	-	Nile	120	3683	4.04	0.06	COM	2017	
Sangoro	Magwagwa	Nile	21	2553	21	0.32	COM	2010	
Kindaruma U3	Magwagwa	Nile	25	2553	21	0.32	COM	2012	
Tana Extension	Magwagwa	Nile	10	2553	21	0.32	COM	2010	
Mutonga	Magwagwa	Nile	60	4537	3.834	0.06	PLN	2016	
low Grand falls	Magwagwa	Nile	60	4537	3.924	0.06	PLN	2016	
Total Ewaso Ngiro	Magwagwa	Nile	180	2739	1.328	0.02	PLN	2017	
Karura	Magwagwa	Nile	56	4049	13.639	0.21	PLN	2016	
				Rwanda					
Mukungwa	Rusumo Falls	Nile	12.5	2000	21	0.32	Historic		
Gihiria	Rusumo Falls	Nile	1.8	2000	21	0.32	Historic		
Gisenyi	Rusumo Falls	Nile	1.2	2000	21	0.32	Historic		
Nyabarongo	Rusumo Falls	Nile	28	5342	3.895	0.06	PLN	2014	
Rukarara	Rusumo Falls	Nile	95	2553	21	0.32	PLN	2014	
Ruzizi II (12MW, shared)	Rusumo Falls	Nile	12	0	21	0.32	Historic		
Ruzizi I (15MW, shared)	Rusumo Falls	Nile	15	0	21	0.32	Historic		
Ruzizi III (48,3MW, shared)	Rusumo Falls	Nile	48.3	2553	21	0.32	0	2018	
Ruzizi IV (95,7MW, shared)	Rusumo Falls	Nile	95.7	2553	21	0.32	0	2019	
				Sudan					
Sennar and Extension	-	Nile	15	2553	21	0.32	Historic		
Roseires	-	Nile	280	2553	21	0.32	Historic		
Kashm El Girba	-	Nile	17.8	2553	21	0.32	Historic		
Jebel Aulia	Gabal Awlia Dam	Nile	28.8	2553	21	0.32	Historic		
Merowe	-	Nile	28.8	2553	21	0.32	Historic		
Bedden	-	Nile	400	2973	0.273	0.00	PLN	2030	
Fula	-	Nile	720	2474	1.112	0.02	PLN	2030	
Lakki	-	Nile	210	2629	6.936	0.10	PLN	2030	
Shukoli	-	Nile	210	2571	3.642	0.05	PLN	2030	
Dagash	-	Nile	284.8	3792	9.82	0.15	PLN	2025	
Kagbar	-	Nile	300	3433	4.513	0.07	PLN	2021	
Low Dal	-	Nile	340	4124	1.522	0.05	PLN	2028	
Sabloka	-	Nile	120	6383	12.503	0.19	PLN	2028	
Shereiq	-	Nile	315	3613	3.559	0.05	PLN	2020	
Rumela	-	Nile	30	8116	13.041	0.20	COM	2013	
				Tanzania					
Mtera	Rumakali	Zambezi	80	2553	21	0.32	Historic		

Kidatu	Rumakali	Zambezi	204	2553	21	0.32	Historic	
Hale	Magwagwa	Nile	21	2553	21	0.32	Historic	
Kihansi	Rumakali	Zambezi	180	2553	21	0.32	Historic	
Pagani Falls	Pangani	Nile	680	2553	21	0.32	Historic	
Nyumba Ya Mungu	Magwagwa	Nile	8	2553	21	0.32	Historic	
Ruhudji	Rumakali	Zambezi	358	1717	3.869	0.01	PLN	2016
Russomo	-	Nile	80	5486	32.021	0.06	PLN	2017
Kakono	-	Nile	53	1962	50.58	0.09	PLN	2025
Songwe Bigupu	Songwe	Nile	34	3638	113.223	0.19	PLN	2017
Songwe Sofre	Songwe	Nile	157	2390	29.045	0.05	PLN	2017
Songwe Manolo	Songwe	Nile	149	2561	35.935	0.06	PLN	2017
Masigira	Rumakali	Zambezi	118	2088	50.569	0.09	PLN	2020
Mpanga	Rumakali	Zambezi	144	2041	44.883	0.08	PLN	2018
Tevete	Rumakali	Zambezi	145	3031	51.218	0.09	PLN	2020
Rumakali	-	Nile	222	2568	35.72	0.06	PLN	2019
Ikondo	Rumakali	Zambezi	340	2181	26.829	0.05	PLN	2019
Stieglers Gorge 1	Rumakali	Zambezi	300	3614	19.311	0.03	PLN	2023
Stieglers Gorge 2	Rumakali	Zambezi	600	644	10.411	0.02	PLN	2023
Stieglers Gorge 3	Rumakali	Zambezi	300	1056	22.331	0.04	PLN	2023
Kishanda	Rumakali	Zambezi	207	1313	21	0.32	PLN	2016
Uganda								
Kiira	-	Nile	200	0	21	0.32	Historic	
Bujagali	-	Nile	250	2553	21	0.32	COM	2011
Nalubaale	-	Nile	380	0	21	0.32	Historic	
Ayago	-	Nile	612	3516	3.5014	0.05	PLN	2018
Isimba	-	Nile	100	3630	3.501	0.05	PLN	2018
Karuma High	-	Nile	700	3990	3.5	0.05	PLN	2019
KIBA	-	Nile	288	2553	21	0.32	PLN	2022
Murchison Falls	-	Nile	750	2211	3.503	0.05	PLN	2037

*COM-committed plants which will come into operation in the designated year, PLN- planned power plants which don't have a start date but are in the design and planning phase

Supplementary Table 14: Existing international transmission capacity[50]

Country 1	**Country 2**	**Capacity (MW)**
Uganda	Kenya	418
	Rwanda	250
	Tanzania	59
Burundi	Rwanda	100
Ethiopia	Sudan	200
	Djibouti	180
DRC	Rwanda	157

Supplementary Table 15: Future planned international transmission capacity[50]

Country 1	Country 2	Capacity (MW)	Earliest
Tanzania	Kenya	1520	2015
	Uganda	700	2023
Uganda	Kenya	440	2023
Ethiopia	Kenya	2000	2016
		2000	2020
	Sudan	1600 x 2	2020
		1600	2025
Egypt	Sudan	2000	2016
		2000*	2020
		2000*	2025
DRC	Rwanda	370	2014
	Burundi	330	2014

* These capacities have not been included in this study, as there existed no literature to confirm their actual plan when this study was conducted

Supplementary note 1

Evaluating the climate resilience of power pool expansion plans

The approach used in this paper does not rank the different plans with a merit order but organises the information in a manner easy for the planning authorities to understand how different plans would perform across a wide range of futures and compare their robustness/resilience [51,52,53]. The methodology has been tested in similar settings related to water management and flood risk assessments involving high levels of uncertainty[54–56]. The methodology considers two decision criteria to select a resilient strategy.

A mini-max criterion evaluates strategies (electricity generation mix) across the entire range of climate futures and identifies an option that minimises the worst-case regret. In this criterion, a matrix of Net present values (NPVs) of the analysed strategies and possible climatic futures is prepared. It is similar to a payoff matrix constructed to evaluate decision outcomes against multiple future scenarios. From the NPV matrix, the maximum NPV for each climatic future is identified. A new matrix is derived by subtracting the NPVs of each strategy (for a designated climate) from the maximum value for the same climate. This new matrix is called the regret matrix. Now the regret of each strategy across the different climates is evaluated, and the maximum value is identified. The most resilient strategy is the one with the smallest value from all the maximum regrets identified in the previous step. This is a high-risk prevention approach and preferred when there can be no real probability assigned to how the climate transpires.

A modified version of a domain criterion that evaluates a strategy with the smallest 75th percentile regret is also utilised in this study. This criterion builds on Starr's domain criterion which uses the same matrix of NPVs. Here we implement a constraint that eliminates extreme outcomes and takes into consideration only the 75th percentile of the regret values for each climate future; hence removing the outlier regret values and thereby making the choice less susceptible to extremes.

In this analysis, we assess seven different climatic projections: six projections from GCM downscaled outputs and one based on the historical climatic pattern. The perfect foresight adaptation (PF) runs are conducted for each of the seven climates, and the optimal strategies are identified. The no-adaptation runs followed the PF runs, where each expansion strategy from the previous stage is run with six other climates to simulate their responses. Therefore, in total there are 49 scenario runs. As part of the process to choose the most resilient climate strategy, we calculated the Net present value (NPV) of all possible combinations, i.e. the perfect foresight adaptation (PF) expansion plan for each climate future is treated as a no-adaptation (NA) strategy for each of the other climate futures to evaluate their performance across the ensemble of climates. The NPV for each of the combinations is presented in Supplementary Table 3. The row header represents the climate numbers, whereas the column headers represent the PF strategy chosen as the fixed rollout plan. Climate 0 and strategy 0 refer to the baseline climate and its perfect foresight strategy. To give an example, the 2nd column refers to the NPV, when the PF strategy for climate 124 is fixed and evaluated across the different climates.

NPVs from Supplementary Table 3 are utilised to calculate the regret for choosing a strategy in each climate future. The difference in the NPV of the assessed strategy to the best performing strategy for that climate is referred to as the regret. The calculated regret for each combination is presented in Supplementary Table 4. Both, the mini-max criterion and modified version of the domain criterion lead us to the same resilient choice - Strategy 137. It is interesting to note that, the chosen strategy is the PF strategy for climate 137, a slightly wetter climate than the baseline.

Supplementary Note 2

Impact of technology learning rates on the generation mix

An important uncertainty that is not explored in this analysis is the impact of a reduction in technology costs (from learning rates) on the power pool's generation mix. To improve our understanding on how implementing learning rates might affect our reference scenario results, we develop a new scenario where Africa specific, learning rates from the 2016-world energy outlook[57] are applied to the technology costs used in the energy model of the EAPP. All the other assumptions remain the same, as in the original reference scenario. Supplementary Figure 21 and Supplementary Figure 22 illustrate the difference in the total installed capacity (GW) and electricity generation (TWh) between the new scenario with technology learning and the original reference (without learning), for the EAPP. Also plotted is the annualised cost of electricity generation in the two scenarios.

We can notice that the results for the total installed capacity are not significantly different until 2020, where the first substantial investments in solar (PV) start to appear. We can notice that the availability of cheap solar power initiates the replacement of new capacity investments, and generation, from natural gas-based power plants. The cost of electricity generation is expected to increase in the beginning as new investments are made in grid-connected and distributed solar PV. In between 2017-2050, the investment in solar technologies (in GW) is three times the capacity, of gas and coal power plants, that it displaces. Supplementary Figure 23 shows the difference in the total installed capacity across the model period, between the two scenarios; it provides an understanding of how much extra (solar) capacity is introduced. Similarly, Supplementary Figure 24 illustrates the difference in electricity generation (TWh) for the same set of scenarios.

As we move forward in the model period, the displacement of gas and coal-based electricity generation is quite evident. This brings down the cost of electricity generation. It is also interesting to notice that almost no new capacity investments and electricity generation from hydropower are replaced. And most of this inexpensive hydropower in the region is traded between countries through transmission networks. This brings us to an important conclusion that implementing cost reductions due to technology learning will replace centralised fossil fuel based electricity generation, but not from hydropower. Of course, the effects of a progressively dry climate could result in higher—non-hydro—renewable penetration, which needs to be explored in detail.

That being said, the electricity trade capacity in the region is essential to mitigate the impact of climate change, and substantial investment in solar PV without adequate electricity storage can result in grid instability. This is evident from the distributed diesel-based electricity generation in the mix, towards the end of the model period. This diesel-based generation is expected to meet the peak demand, which was, erstwhile, satisfied by the gas power plants. Hence, massive investment in solar power without proper storage could result in higher costs. This is evident from the downward trend in the cost of electricity generation towards the end of the modelling period in Supplementary Figure 24.

Supplementary note 3

To capture the effects of uncertain precipitation and temperature patterns on the Nile River basin and its constituent countries, a framework involving two types of modelling methodologies is developed. A detailed systems model of the entire Eastern African Power Pool's (EAPP) electricity infrastructure, which included the electricity generation mix of all the constituent countries modelled in detail and a water systems management model of the Nile River basin. The two modelling frameworks are soft linked to assess the climate resilience of the water and energy infrastructure, in East Africa, to a changing climate.

Water management (Balance) model of the Nile River Basin

The Water Evaluation and planning tool (WEAP- http://www.weap21.org/) developed by the Stockholm Environmental Institute (SEI) is used to model the entire Nile River Basin. WEAP is a hydrological water systems management model which has been refined over the past 20 years and used by more than 10,000 registered users from over 170 countries. The Nile River Basin, constituting the Eastern Nile (Ethiopian Highlands and above) and the Nile equatorial lakes (the region surrounding Lake Victoria) are divided into more than 150 sub-catchments. To maintain consistency and for validation purposes, the water model is calibrated against historical stream flows for different measurement points across the basin including the two most significant tributaries; the White and the Blue Nile. Some critical inputs to the model, but not restricted to, include the following:

- Detailed population data (current and future estimates)
- Technical characteristics of all major hydropower plants (both existing and future) in the East African region
- Major irrigated crops in each of the ten countries
- State-specific irrigation master plans
- Environmental flow restrictions along the River Nile

An elaborate description of the water model (WEAP) of the Nile River basin, developed for this study can be accessed from Huber-Lee et al.[58].

Long-term electricity sector expansion model of the Eastern African Power Pool (EAPP)

A long-term power sector expansion model of the Eastern African power pool (EAPP) is developed using the Open Source Energy Modelling System (OSeMOSYS- http://www.osemosys.org/). OSeMOSYS is one of the few open-source energy systems optimisation frameworks that are currently being used to develop pathways for energy planning on national, continental and global levels[59, 60–62]. It is a dynamic, bottom-up, multi-year energy system model applying linear optimisation techniques. The modelling framework consists of demand projections and a database of power supply technologies that are characterised by economic, technical and environmental parameters, and information regarding the existing capital stock and its remaining lifespan. The model is restricted by constraints used to reflect, amongst others, operational requirements, governmental policies, or socioeconomic realities.

An OSeMOSYS model for the EAPP is developed for this assessment. The model produces an optimal electricity generation mix, for each modelled year, whose cost has been optimised to be the lowest over the entire period of the study. Like other optimisation models, OSeMOSYS assumes a perfect market with perfect competition and foresight.

Model Structure and Demand Projections

The OSeMOSYS model of the EAPP, henceforth referred to as the energy model, is similar to the model developed by Taliotis et al.[59]; involves a set of demand projections and an extensive set of supply technologies to satisfy the demand. All energy carriers in the model are categorized as fuels and all energy conversion infrastructure ranging from power plants, pipelines and refineries are grouped as technologies. To capture the impact of decentralised power supply options—which can present a competitive alternative to grid-connected and large-scale power supply options—the electricity demand is split into three categories: Urban, Rural, and Industrial. Each demand category has exclusive supply chains linking stand-alone and grid-connected power supply options to the final useful electricity demand. A simplified representation of the reference energy system (RES), a schematic of all the energy chains, technologies and fuels is illustrated in Supplementary Figure 25.

The Final electricity demand is the primary driver to be satisfied in each of the time-periods. The model is forced to invest in infrastructure along the individual energy chains accordingly. The final electricity demand for each country in the Power Pool is calculated and represented separately. The demands are computed using past correlations between final electricity consumption and country-specific economic and social indicators (GDP, Population)[63]. These base values are projected over the model period to estimate the final electricity demand. Country-level statistics on rural and urban population, electrification rates and sector-specific GDP and energy intensities are used to split the demand into the following categories. (a)Heavy industry: which connects to generation at a high voltage level and has the lowest transmission and distribution losses. (b)Urban residential: linked to sources of generation via a more extensive transmission and distribution system with associated losses. (c) Rural residential: which requires, even more, transmission and distribution infrastructure (higher losses).

With electricity demand being the primary driver, the model is susceptible to the changes in the profile and magnitude of sectoral demands. There exist an extensive set of uncertainties related to the demand projections regarding, country-specific social and economic indicators and sudden industrial sector growth stimulated by a new and cheap source of electricity (GERD dam in Ethiopia). Hence, the results need to be analysed with a grain of salt.

Temporal Resolution and Load Curves

For each of the final useful electricity demands, certain energy carriers need to be produced upstream in the energy chain; hence, it is mandatory to define how much demand needs to be satisfied in a defined unit of time. These splits, also called time slices/year-splits, are of paramount importance in optimisation models and are dependent on factors like, but not restricted to, availability of natural resources (solar, hydro, wind), peak demand periods and storage options[64].

Since the focus is to integrate the water balance and electricity expansion models and assess the uncertainty in the availability of water for energy infrastructures (hydropower), both long-term and seasonal climate variations had to be considered. Hence, each year is broken down into 12 month periods; additionally, each month is divided in two day-types (weekdays and weekends), and each specific day-type is further split into two parts to represent periods of low and high demand. This division led to 48

splits in a year. The share of electricity load falling under each of the computed time steps is calculated and input to the model. Supplementary Table 7 and Supplementary Table 8 provide the necessary time slice definitions used in this study.

Country representations and cross-border links in the model

Cross-border transmission links play a critical role in distributing the energy resources that are spread across different countries. A country like Egypt with plenty of natural gas reserves and countries like Uganda and Ethiopia with high renewable—though only seasonal—potential can complement each other to improve access to electricity in the entire power pool. In the EAPP model, the countries are linked using inter-country trade connectors that represent the cross-border electricity transmission networks. These transmission lines are categorized as technologies that link two parallel energy chains between two neighbouring countries. They transfer electricity from Country A to Country B and vice-versa by a two-way connection.

Electricity Generation options

The EAPP model considers a comprehensive set of electricity generation options in the optimization routines. They are broadly classified into grid-connected and stand-alone systems. They are namely:

- Distributed, diesel fired internal combustion engines that can serve Industrial, urban or rural demand
- Centralized diesel systems connected to the transmission network
- Heavy fuel oil-fired power plants connected to the transmission network
- Open cycle gas turbines connected to the transmission network
- Combined cycle gas turbines connected to the transmission network
- Large hydro (dam or run-of-river) connected to the transmission network
- Small or mini-hydro facilities (below 10 MW) that can supply the distribution network
- Onshore wind facilities connected to the transmission network. Two options are included here; one with an average capacity factor of 25% and another with 30%
- Biomass-fired plants connected to the transmission network
- Large-scale solar PV facilities connected to the transmission network
- Rooftop solar PV facilities. Three options are modelled here; without storage, with one kWh battery or with two kWh battery
- Solar CSP connected to the transmission network. CSP technologies are modelled in three alternatives; without storage, with thermal storage or with gas co-firing

It should be noted that the fossil fuel technologies are grouped based on the fuel used, the technology used and size, but the large hydropower plants are represented as individual technologies in the model to capture the seasonal variability of water at each plant as an input from the water model.

Assumptions in the models

The assumptions for technical and economic parameters used in the model are provided in Supplementary tables 5-15. A discount rate of 5% is used for the study. This rate is used considering that the study is conducted with a focus on the perspective of a social planner and not a private investor[65]. Furthermore, the study assessed longer periods until 2050. Hence the decision.

Supplementary note 4

The need for site-specific large hydropower representation

We represented the large hydropower plants, in the model, taking a site-specific approach rather than combining them into one single technology option as followed in other similar studies [59]. The reasons behind this disaggregation are explained below.

- The difference in costs: The capital costs of most of the power plants vary depending on the location, type and size
- Other site-specific competing uses: Some reservoirs are shared between different end uses of water—domestic usage, irrigation and electricity generation
- Climate variability: hydropower plants in the same region/country could be part of a different sub-basin of the Nile River; hence, a variation in the climate and land use induced runoff could be different.
- We can notice in Supplementary Figures 25-28, the variation in hydropower generation across different climates. These are snapshots for years 2020, 2030, 2040 and 2050; each figure plots the monthly generation variability across all the seven climates emphasising the need for individual representation to derive insights that are more accurate.

Supplementary note 5

Selection of climate futures

Assigning a probability to a future climate to turn out a certain way has never been a simple task for climate scientists[66]. The precipitation and temperature patterns projected for the East African region face the same issue[67]. To address the high level of uncertainty, an ensemble of climate projections is derived using the Bias-corrected Spatial Disaggregation method (BCSD) using results from two classes of climate models: namely the CMIP5 and CMIP3 from the Assessment Report 5 (AR5) and 4 (AR4) respectively of the IPCC. Historical Baseline used for this study used the data from the Terrestrial Hydrology research group at Princeton University, organised in a 0.5deg X 0.5deg resolution for the period from 1948-2008. The BCSD process resulted in 121 different climate futures spanning over three emission scenarios from the AR4 namely: A1, A1B, A2; and two Representative concentration pathways from AR5 namely: RCP4.5 and RCP8.5.

Since running the water and the energy model over 121 different futures, with monthly time resolution for the water model and a higher temporal split for the latter is computationally intensive, a representative set of six climate futures is chosen out of the 121 available options. The 121 climate futures are ranked based on their Climate Moisture Index (CMI) value averaged over the modelling period, and a simple algorithm is developed to choose six cases, which spanned over different percentile ranges and represented a good sample of the range of precipitation and temperature outcomes inferred from the 121 climates. The algorithm[1] involved the following criteria.

- Includes an extreme wet and dry future for each of the seven basins. We define an extreme future as one outside the 5%-95% range for the full ensemble of climates. This criterion ensures we consider a stressing future for each basin.
- Includes futures with extremes for several basins. This criterion ensures that we consider futures that stress multiple basins simultaneously.
- Include a future close to the average over the full ensemble. This criterion ensures that we have an appropriate comparison for the extreme cases.
- Includes a significant number of CMIP5 runs, and derives from a mix of medium and high emissions scenarios. This criterion helps to ensure that the set of futures includes the most recent climate information and provides some information regarding how alternative emission trajectories might affect infrastructure investment plans.

Supplementary Table 2 has the numbering and details of the selected climate model outputs.

Supplementary note 6

Integrating the energy model (OSeMOSYS) with the water model (WEAP)

The framework developed for this study draws inspiration from various methodologies discussed in the literature [68, 69, 70] to calculate the cost of adapting the energy and water infrastructures to climate change. This methodology involves developing a water systems management model of the Nile river basin and soft linking it with a detailed electricity sector expansion model of the EAPP and running them through a set of carefully chosen climate scenarios. The starting point is to develop a baseline in both the energy and water models. For this study, the development plan from the Program for Infrastructure Development in Africa (PIDA) is used as a reference for existing and planned energy and water infrastructures. In addition to PIDA, country level irrigation and water infrastructure master plans and electricity expansion master plans from the power pool are appended to create an extensive set of existing and planned infrastructure; both committed projects under construction and those just under the initial phase of planning are included. Henceforth this newly developed plan, referred to as PIDA+ is used as a baseline in all the models.

The information flow between the energy and water models are managed by harmonising techno-economic characteristics of the hydropower plants in the respective frameworks. This process is challenging due to the dissimilar features of the two models. OSeMOSYS is an optimisation model that considers the best combination of electricity generation options and correspondingly dispatches power to meet demand and minimise the total system cost. WEAP is not an infrastructure expansion model. It is a versatile water accounting tool where the infrastructure is input from the analyst rather than as output. WEAP is a geo-referenced and topology-dependent tool. OSeMOSYS, on the other hand, is an abstraction of the electricity generation options in the country with detailed techno/economic representation. The OSeMOSYS model contains a more extensive set of hydropower plants than that are not included in the WEAP river basin models. It contains hydropower plants outside the Nile Basin, which are not part of the water model but are critical for the given country's electricity infrastructure. Considering that the objective of the study is to investigate the impact of climate change in the region through the changes in water availability on a facility level, this is a potential challenge. It is overcome by introducing a proxy procedure to ensure that the capacity factors for hydropower plants are consistent across the different climate futures. The capacity factors for the plants outside the Nile basin are proxied from the nearest hydropower plant existing inside the basin.

To overcome these challenges, the water and energy frameworks are integrated using a specific multi-stage data exchange protocol through an intermediary, Matlab based two-tier optimisation tool. Using economic evaluations of the benefits of specific infrastructure options, the algorithm adjusts first the irrigation investments and second the characteristics of shared hydropower infrastructure to maximise basin-wide net economic benefits. To do so, the water systems model is run repeatedly using a pattern search algorithm that iteratively alters the irrigation and hydropower investment characteristics until an optimal solution is found. These changes offer new sets of water availability for the different facilities in the EAPP model.

The iteration between the two modelling frameworks began with the water model of the Nile river basin due to the complex representation of water requirements in the model. The water model estimated monthly water availability at each hydropower plant in the model, and the results from the intermediary

Matlab based optimisation tool are used to adjust the capacity of the hydropower stations if they are technically and economically feasible. Power plant level capacity changes and monthly variations in water availability are then communicated to the EAPP model through a seamless soft linking procedure using plant-specific capacity factors. This process is iterated for each climate run to converge towards an optimal hydropower infrastructure within the technically feasible boundaries of possible hydropower configurations for each site.

The water availability is translated into capacity factors for each hydropower plant as follows.

C_f Plant specific Capacity Factor

$\forall\ i, j \in \{technologies\} \times \{time\ slices\} \quad C_f^{i,j} = \frac{E_i}{C_i * \Delta t_j}$ **eq.1**

E_i Energy generated by the power plant i

Δt_j The corresponding duration (each month of each year)

C_i Power plant capacity

The capacity factors are calculated for each hydropower plant, time-slice, and climate scenario. Since the water availability for hydropower plants is calculated on a monthly basis in the water model, the capacity factors, for each power plant, must reflect the modality and be the same for each month. However, the power pool model had a better temporal resolution; having four splits in each month. Hence, to maintain coherence, the four time-slices of each month are made to associate with the same hydro capacity factor.

Supplementary references

48. IRENA. *West African Power Pool: Planning and Prospects for Renewable Energy*, http://irena.org/DocumentDownloads/Publications/WAPP.pdf, (IRENA, 2013).

49. U.S. Energy Information Administration (EIA). (2014), http://www.eia.gov/countries/regions-topics.cfm?fips=EM, (2014)

50. SNC lavalin international inc & Parsons Brinckerhoff. EAPP Master Plan, http://eappool.org/the-master-plan-2011, (2011)

51. Kenneth M. Strzepek, Raffaello Cervigni, Rikard Liden, Robert Lempert, Mark Howells, David Purkey, Brent Boehlert & Yohannes Gebretsadik. Chapter 2: Methodology. in *Enhancing the Climate Resilience of Africa's Infrastructure : The Power and Water Sectors*, https://openknowledge.worldbank.org/handle/10986/21875, (The World Bank, 2016).

52. Lempert, R. J. & Collins, M. T. Managing the risk of uncertain threshold responses: comparison of robust, optimum, and precautionary approaches. *Risk Anal. Off. Publ. Soc. Risk Anal.* **27**, 1009–1026 (2007).

53. Hall, J. W. *et al.* Robust Climate Policies Under Uncertainty: A Comparison of Robust Decision Making and Info-Gap Methods. *Risk Anal.* **32**, 1657–1672 (2012).

54. Kasprzyk, J. R., Nataraj, S., Reed, P. M. & Lempert, R. J. Many objective robust decision making for complex environmental systems undergoing change. *Environ. Model. Softw.* **42**, 55–71 (2013).

55. Lempert, R. J. & Groves, D. G. Identifying and evaluating robust adaptive policy responses to climate change for water management agencies in the American west. *Technol. Forecast. Soc. Change* **77**, 960–974 (2010).

56. Hallegatte, S. S., Ankur Lempert, Robert Brown, Casey Gill, Stuart. *Investment Decision Making under Deep Uncertainty - Application to Climate Change*. (The World Bank, 2012). doi:10.1596/1813-9450-6193

57. IEA. *World Energy Outlook 2017*. (OECD Publishing, Paris/International Energy Agency, Paris, 2017), https://www.oecd-ilibrary.org/energy/world-energy-outlook-2017_weo-2017-en, (2017)

58. Annette Huber-Lee, Stephanie Galaitsi, Casey Brown, & Abdulkarim Seid, Denis Hughes, and Brian Joyce. Reference Investment Scenario; Chapter 4 ; WEAP model Development. in *Enhancing the Climate Resilience of Africa's Infrastructure : The Power and Water Sectors*, https://openknowledge.worldbank.org/handle/10986/21875, (The World Bank, 2016).

59. Taliotis, C. *et al.* An indicative analysis of investment opportunities in the African electricity supply sector — Using TEMBA (The Electricity Model Base for Africa). *Energy Sustain. Dev.* **31**, 50–66 (2016).

60. Pinto de Moura, G. N., Loureiro Legey, L. F., Balderrama, G. P. & Howells, M. South America power integration, Bolivian electricity export potential and bargaining power: An OSeMOSYS SAMBA approach. *Energy Strategy Rev.* **17**, 27–36 (2017).

61. Taliotis, C., Howells, M., Bazilian, M., Rogner, H. & Welsch, M. Energy Security prospects in Cyprus and Israel: A focus on Natural Gas. *Int. J. Sustain. Energy Plan. Manag.* **3**, 5–20 (2014).

62. Welsch, M. *et al.* Modelling elements of Smart Grids – Enhancing the OSeMOSYS (Open Source Energy Modelling System) code. *Energy* **46**, 337–350 (2012).

63. Saadi, N., Miketa, A. & Howells, M. African Clean Energy Corridor: Regional integration to promote renewable energy fueled growth. *Energy Res. Soc. Sci.* **5**, 130–132 (2015).

64. Poncelet, K., Delarue, E., Six, D., Duerinck, J. & D'haeseleer, W. Impact of the level of temporal and operational detail in energy-system planning models. *Appl. Energy* **162**, 631–643 (2016).

65. Goulder, L. H. & III, R. C. W. *The Choice of Discount Rate for Climate Change Policy Evaluation*. (National Bureau of Economic Research, 2012). doi:10.3386/w18301

66. Knutti, R. & Sedláček, J. Robustness and uncertainties in the new CMIP5 climate model projections. *Nat. Clim. Change* **3**, 369–373 (2013).

67. Kent, C., Chadwick, R. & Rowell, D. P. Understanding Uncertainties in Future Projections of Seasonal Tropical Precipitation. *J. Clim.* **28**, 4390–4413 (2015).

68. Lucena, A. F. P. *et al.* Climate policy scenarios in Brazil: A multi-model comparison for energy. *Energy Econ.* **56**, 564–574 (2016).

69. Ciscar, J.-C. & Dowling, P. Integrated assessment of climate impacts and adaptation in the energy sector. *Energy Econ.* **46**, 531–538 (2014).

70. de Lucena, A. F. P., Schaeffer, R. & Szklo, A. S. Least-cost adaptation options for global climate change impacts on the Brazilian electric power system. *Glob. Environ. Change* **20**, 342–350 (2010).
